# The dynamics of free and phosphopeptide-bound Grb2-SH2 reveals two dynamically independent subdomains and an encounter complex with fuzzy interactions

**DOI:** 10.1038/s41598-020-70034-w

**Published:** 2020-08-03

**Authors:** Karoline Sanches, Icaro P. Caruso, Fabio C. L. Almeida, Fernando A. Melo

**Affiliations:** 1 Institute of Medical Biochemistry – IBqM, Federal University of Rio de Janeiro, Rio de Janeiro, Brazil; 2 National Center for Structural Biology and Bioimaging (CENABIO)/National Center for Nuclear Magnetic Resonance (CNRMN), Federal University of Rio de Janeiro, Rio de Janeiro, Brazil; 3 Multiuser Center for Biomolecular Innovation (CMIB), Department of Physics, São Paulo State University (UNESP), São Jose do Rio Preto, São Paulo Brazil

**Keywords:** Proteins, Oncogene proteins, Structure determination, NMR spectroscopy, Molecular biophysics, Biochemistry, Biophysics

## Abstract

The growth factor receptor-bound protein 2 (Grb2) is a key factor in the regulation of cell survival, proliferation, differentiation, and metabolism. In its structure, the central Src homology 2 (SH2) domain is flanked by two Src homology 3 (SH3). SH2 is the most important domain in the recognition of phosphotyrosines. Here, we present the first dynamical characterization of Grb2-SH2 domain in the free state and in the presence of phosphopeptide EpYINSQV at multiple timescales, which revealed valuable information to the understanding of phophotyrosine sensing mechanism. Grb2-SH2 presented two dynamically independent subdomains, subdomain I involved in pY recognition and subdomain II is the pY + 2 specificity pocket. Under semi-saturated concentrations of pY-pep we observed fuzzy interactions, which led to chemical exchange observed by NMR. This information was used to describe the encounter complex. The association with pY-pep is dynamic, involving fuzzy interactions and multiple conformations of pY-pep with negative and hydrophobic residues, creating an electrostatic-potential that drives the binding of pY-pep. The recognition face is wider than the binding site, with many residues beyond the central SH2 binding site participating in the association complex, which contribute to explain previously reported capability of Grb2 to recognize remote pY.

## Introduction

Cell survival, control, proliferation, differentiation, and metabolism are mediated by a variety of well-orchestrated series of events. In this context, the growth factor receptor-bound protein 2 (Grb2) has demonstrated to be a key factor in regulating many cellular events. Grb2 is not an enzyme, instead, its multiple domains and flexible linkers provide the ability to bind to multiple partners in the cell. Grb2 is a pivotal intermediate in the communication between the cell-surface and downstream signaling^1^. Recently, Grb2 has been reported to have equilibrium between monomeric and dimeric states, which is critical for the activation and regulation of the Ras/mitogen-activated protein kinases (MAPKs) pathway^2^. The plasticity of Grb2 is essential for its biological function. It is widely known that the deregulation of MAPK pathway leads to many diseases, including cancer and developmental defects^3–6^.

Grb2 is composed of a central Src homology 2 (SH2) domain flanked by two Src homology 3 (SH3), a N- and C-terminus. The SH3 domain is well-known to recognize proline-rich sequences with the PxxP motif, while SH2 is the most important domain in the recognition of phosphotyrosines (pY)^7–9^. The SH2 has approximately 100 amino acid residues in globular modules with a well-conserved structure of a central antiparallel β-sheet flanked on each side by an α-helix. A remarkable point in the SH2 domains is the presence of two distinct binding pockets for the phosphopeptide recognition, where the pocket I (site I) is responsible for the pY binding to two conserved residues, RβB5 (R186) and HβD4 (H107), and the pocket II (site II) for the pY + 1, pY + 2 or even pY + 3 recognition^8,10^. Site II accounts for the specificity of the interaction. The phosphopeptide binds to SH2 as an extended linear conformation at the domain surface that spans from one α-helix to the other. An exception for this linear conformation is the interaction with the SH2 domain of Grb2, in which the W121 (site II) is a barrier, hindering the pY + 3 interaction. Grb2-SH2 preferentially selects pY + 2 asparagine sequences^11,12^.

Grb2-SH2 domain is able to form a domain-swapped dimer^13–16^, in which both α-helix 3 (α3) and part of the loop are involved in a major conformational change. The α3 opens and associates with the adjacent subunit of the dimer. The domain-swapped dimer is able to bind phosphopeptides with different affinities when compared to the monomeric state, sometimes with higher affinity and others with lower affinitiy^14,15^, contributing as another degree of freedom for Grb2 plasticity and ability of regulation. The biological role of the swapped domain is not well-known and there is no description of a cellular event with the participation of the swapped Grb2 dimer. The only structure available of the full-length Grb2 is not swapped^17^.

Here we present the first description of the dynamics of Grb2-SH2 domain. We verified that the Grb2-SH2 domain presents two dynamically independent subdomains, one of them in fast exchange regime (subdomain I) and other in intermediate exchange (subdomain II). The thermodynamic profile for a two-state conformational equilibrium of subdomain II in intermediate exchange regime was evaluated. We also evaluated the influence of Grb2-SH2 dynamics in the recognition of endothelial growth factor receptor (EGFR) derived phosphopeptide EpYINSQV (pY-pep) in a saturated and semi-saturated concentration. We characterized a recognition mechanism that involves the formation of an encounter complex as “binding intermediates”. Lastly, we demonstrated that the association of pY-pep to Grb2-SH2 is fuzzy and dynamic, which is essential for the molecular recognition of phosphotyrosines, where many residues beyond the central Grb2-SH2 binding site participates.

## Results

**Figure 1:**
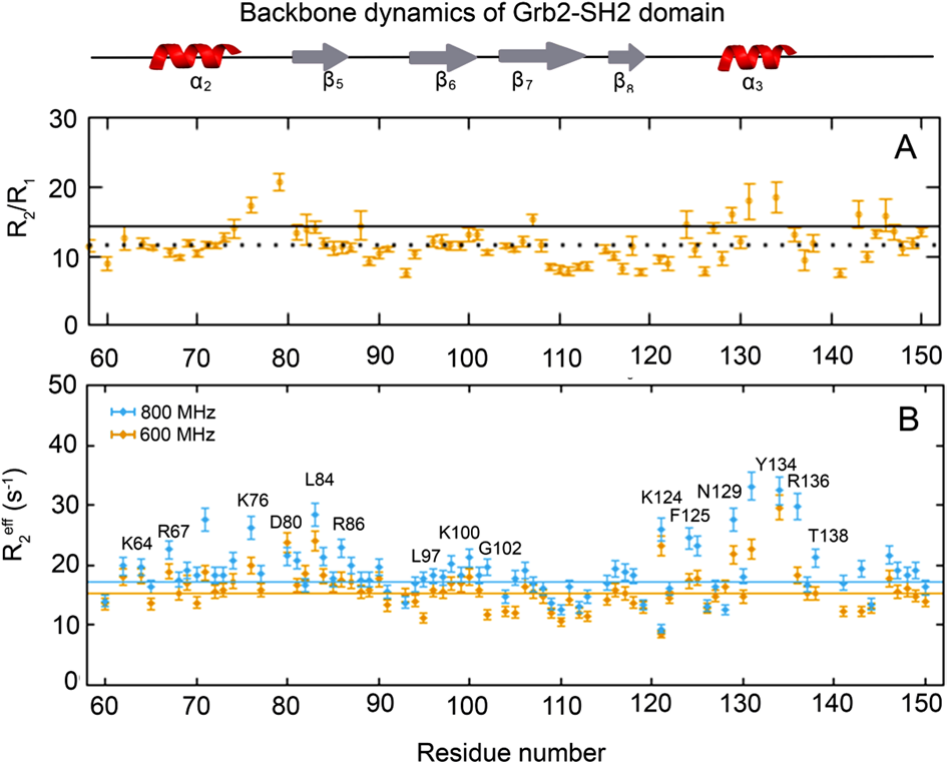
(**A**) Summary of R_2_/R_1_ at 18.8 T (800 MHz) and the (**B**) ^15^N CPMG-RD R_2_^eff^ at 66.7 s^-1^ at 14.09 (600 MHz, orange) and 18.8 T (800 MHz, blue) as function of the residue number, acquired at 25 °C. The lines represent the average R_2_^eff^ for both fields. In **A**, the dotted line represents the average R_2_/R_1_ value and the full line the average value plus one standard deviation. The residues above the average lines are in conformational exchange and are labeled in **B**. There are two main regions in conformational exchange, subdomain I which involves α2 and β5, and subdomain II, at α3. These data are complemented by R_1_, R_2_ and ^1^H-^15^N heteronuclear NOE (Figure S2) and by ^15^N CPMG-RD experiments at 5, 10, and 17 °C (Figures S3, S4, S5, and S6).

Our main purpose is the measurement of the dynamic properties of Grb2-SH2 domain and its role in the binding of pY-pep. This is the first description of the dynamics of Grb2-SH2 domain in the presence and absence of phosphopeptide. We chose the EGFR derived phosphopeptide EpYINSQV (pY-pep) because it leads to the dissociation of the Grb2, as described by Yuzawa et col. (2001)^18^. We measured the ^15^N nuclear spin relaxation parameters R_1,_ R_2_ and ^1^H-^15^N-heteronuclear NOE (Figure S1), which showed that Grb2-SH2 domain is mostly rigid, displaying only a few residues involved in thermal motion (pico- to nanosecond timescale). They are mainly in the loop between α2 and β5. The most evident feature was the presence of an increased value of the R_2_/R_1_ ratio (Fig. 1A) in two regions: the first involving residues in α2, loop α2/β5 and β5, and the second in the loop β8/α3 and α3. We assigned ^19^ (BMRB ID 27,781) and calculated the structure of the Grb2-SH2 domain at pH 7.0 (PDB_id 6VK2) using CS-Rosetta^20^ with the ambiguously and unambiguously assigned NOEs derived from Aria/CNS (Figure S2, Table S1). A detailed description of the structure calculation is in the supplementary material.

**Figure 2:**
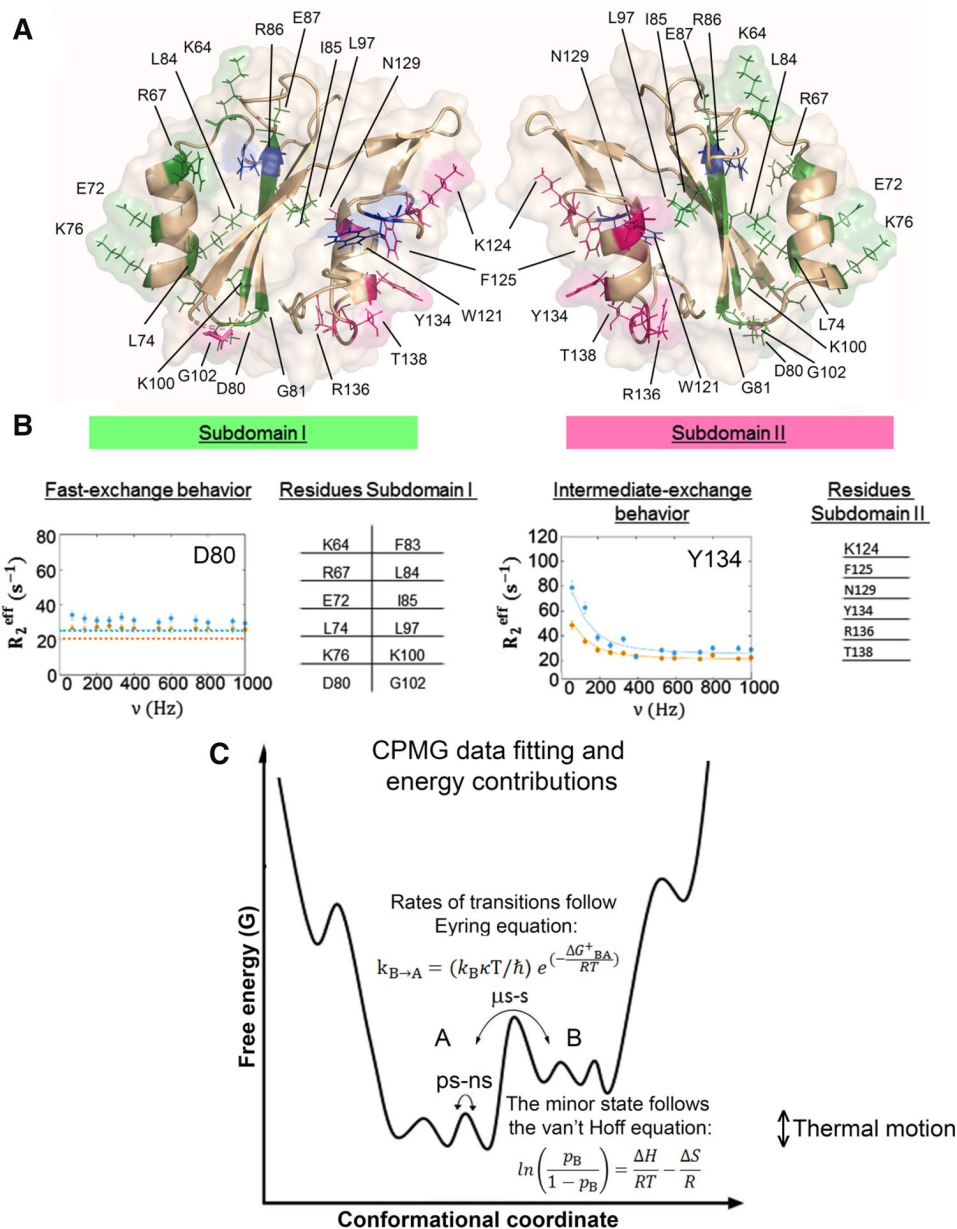
Summary of the regions in conformational exchange of Grb2-SH2 domain obtained from ^15^N CPMG-RD experiments at 5, 10, 17 and 25 °C. (**A**) There are two dynamically independent subdomains. The residues comprising the subdomain I (green) are in fast conformational exchange. The residues comprising the subdomain II (pink) are in intermediate conformational exchange. For reference, the R86 and W121 are colored in blue, evidencing the two subsites for molecular recognition of the pY-pep. (**B**) Typical RD profile of the residues in fast exchange regime observed for subdomain I, represented by D80. The table shows all the residues in fast exchange. Typical RD profile of the residues in intermediate exchange regime observed for subdomain II, represented by Y134. The table shows all the residues in intermediate exchange at subdomain II. The dotted lines represent the exchange-free R_2_^eff^ for each field (**C**) Free energy landscape of a protein undergoing conformational exchange between two states. A is the major conformational state while B is the minor state. For the fitting of the RD profiles of the residues in intermediate exchange using Bloch-McConnell equation, we obtained the population of the minor state (p_B_), the exchange rate (k_ex_ = k_AB_ + k_BA_) and the chemical shift difference between major and minor state (Δω). Using van´t Hoff equation for fitting p_B_ as a function of temperature we obtained the thermodynamic parameter at equilibrium (ΔG, ΔH, ΔS, Fig. 3). Using Eyring equation for fitting k_AB_ or k_BA_ as a function of temperature we obtained the thermodynamic parameter at the transition state (ΔG^‡^, ΔH^‡^, ΔS^‡^, Fig. 3).

These regions are in conformational exchange, and to get more insights into the dynamics we measured the ^15^N CPMG-RD at four temperatures and two fields (Figures S3, S4, S5, and S6). The CPMG-RD data (Fig. 1A) confirmed the observation of conformational exchange (increased R_2_/R_1_ ratio, Fig. 1B). The residues in the first region are at the site I, the phosphotyrosine (pY) recognition site and the residues in the second region are at the site II, the pY + 2 recognition site (Fig. 2A).

The analysis of the CPMG-RD curves showed that the residues in conformational exchange at site I are in fast exchange regime, while the residues at site II are in intermediate exchange regime (Figures S3, S4, S5, and S6). Although they belong to the same domain, they are dynamically independent. Grb2-SH2 has two dynamically independent subdomains: subdomain I (Fig. 2A, residues in green) for the residues in fast exchange involved in recognition site I, and subdomain II (Fig. 2A, residues in pink) for the residues in intermediate exchange involved in the recognition site II.

For the dynamic characterization of subdomain I, we looked for residues with no dispersion, but displaying R_2*eff*_ above exchange-free R_2*eff*_∞. The most likely value R_2*eff*_∞ is represented by the dotted line in Figs. 1B and 2B, which represents an expected average value of R_2*eff*_ for residue without exchange contribution^21^. The typical plot of ^15^N CPMG data observed for fast exchange regimes is where all the points in a flat profile are above the dotted line, meaning that much larger values of ν_CPMG_ would be necessary to refocus the exchange contribution to R_2*eff*_∞ (Fig. 2B). For details, see Figures S3, S4, S5, and S6.

As subdomain II presents residues in intermediate exchange regime, it was possible to characterize its thermodynamic profile for a two-state conformational equilibrium. We fitted the ^15^N CPMG-RD experiments at multiple temperatures using the Bloch-McConnell equation, which describes the evolution of the magnetization in a two-state exchange^22,23^. The Table S1 shows that the two-state exchange regime describes well the dynamics of subdomain II. To evaluate the quality of fitting and validity of the two-state exchange model we compared the statistical parameters (χ^2^ and degrees of freedom, DF) of individual fits for each residue, global fit at each temperature, and the global fit at all temperatures in two situations: (1) without any constrains; and (2) imposing Arrhenius linearity for the transition state and van’t Hoff’ linearity for the equilibrium between major and minor conformational states^24^. Since both global and individual fittings, χ^2^ < DF (global) and Σχ^2^ < ΣDF (individual), the global fitting is consistent with the two-state models. A more detailed description of the analysis is in the supplementary information.

**Figure 3:**
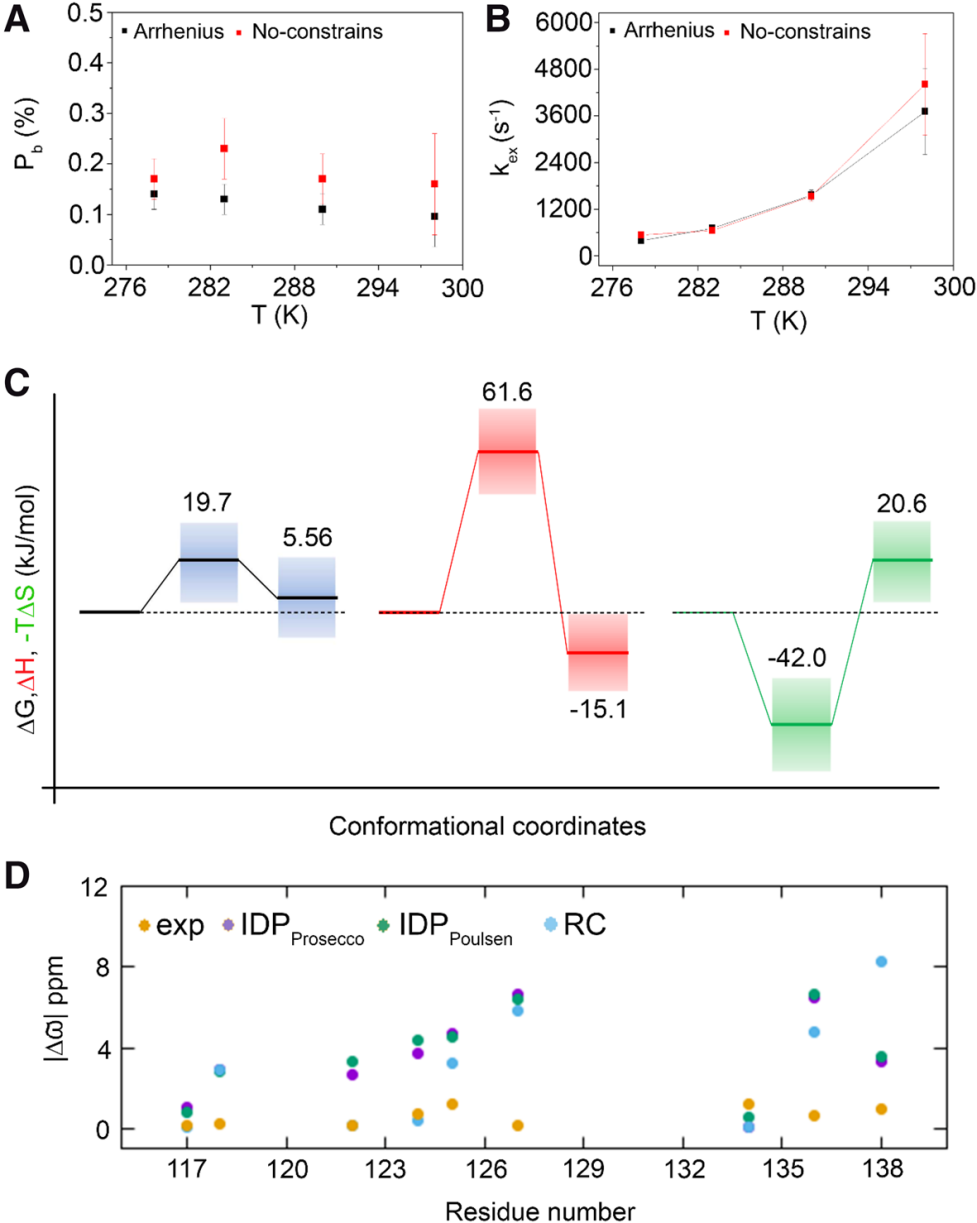
Energy landscape of subdomain II of Grb2-SH2 domain. Thermodynamic parameters of equilibrium and transition state from the global fitting using the Bloch-McConnell equation of ^15^N CPMG-RD experiments at several temperatures. (**A**) The population of the minor state (p_B_) and (**B**) the exchange constant (k_ex_) as function of the temperature. The black and red squares denote the global fitting at all temperatures (no-constrains) and imposing the linearity of Arrhenius and van´t Hoff equations, respectively. (**C**) Conformer A is the major state while B is the first thermally accessible conformational state (minor state). ∆G is in black, ∆H is in red, and − T∆S is in green. Note that the minor conformational state is enthalpically favorable. The thermodynamic parameters obtained from p_B_ and k_ex_ at individual temperatures can be found in Table S1.

The residues K124, F125, Y134, R136, and T138 presented a good ^15^N CPMG-RD profile. Because of the low number of residues in intermediate conformational exchange at subdomain II (Figures S3, S4, S5, and S6), there was a poor convergence of the population of the minor state (p_B_) for the global fitting at each temperature. Conversely, the global fitting at all temperatures and especially the constrained global fitting enabled a good convergence of p_B_. We obtained a good convergence for exchange constant (k_ex_) in all situations. Figure 2C summarizes how to obtain the free energy landscape of a protein from the parameters obtained for the RD profiles. We used van´t Hoff equation to fit p_B_ as a function of temperature and obtain the thermodynamics of equilibrium, and Eyring equation to fit k_AB_/k_BA_ as a function of temperature to obtain the thermodynamics of the transition. These data enabled to obtain the thermodynamic parameters (∆G, ∆H, and ∆S) for the equilibrium and transition state (Fig. 3).

Figure 3A shows the p_B_ and Fig. 3B the k_ex_ for global fitting at all temperatures without and with constraints. The increase in temperature led to a small decrease in p_B_ and an increase in k_ex_. This behavior reflected in the thermodynamic profile, which is observed in Fig. 3C.

The minor state (Fig. 3C) is enthalpically favorable and entropically unfavorable at 298 K. This is typical behavior of a conformational fluctuation involving the exposure of hydrophobic residues to the solvent^24^. Knowing that the conformational exchange involving loop β8/α3 and α3 may be associated with the observation, in several crystal structures, of a domain swap involving α3, which opens to interact with the adjacent subunit to form the swapped dimer^13–16^. The question that remains is whether the minor state is dimeric or monomeric. To answer this question, we acquired the ^15^N-CPMG-RD profiles at 278 K in a lower concentration (120 µM, ~ 2.5 × diluted). These profiles did not have significant changes (Figure S7), meaning that there was not the concentration-dependence expected for an oligomer. We concluded that the observed conformational exchange does not reflect a dimerization of the minor state nor domain swapping. Instead, the minor state is a result of conformational fluctuations of α3 in the monomeric state and related to the pY + 2 recognition.

To get insights on the structural changes at the minor state, we analyzed the differences in the chemical shifts (Δϖ) between the major and minor conformational states and compared with predicted values for the random coil^25^ and the intrinsically disordered domain (Prosecco^26^ and Poulsen^27^, Fig. 3D). The observed Δϖ´s are small when compared to the expected values for the unfolding/disordering of α3. The minor state is probably a result of the repositioning of α3, exposing hydrophobic residues to the solvent.

**Figure 4:**
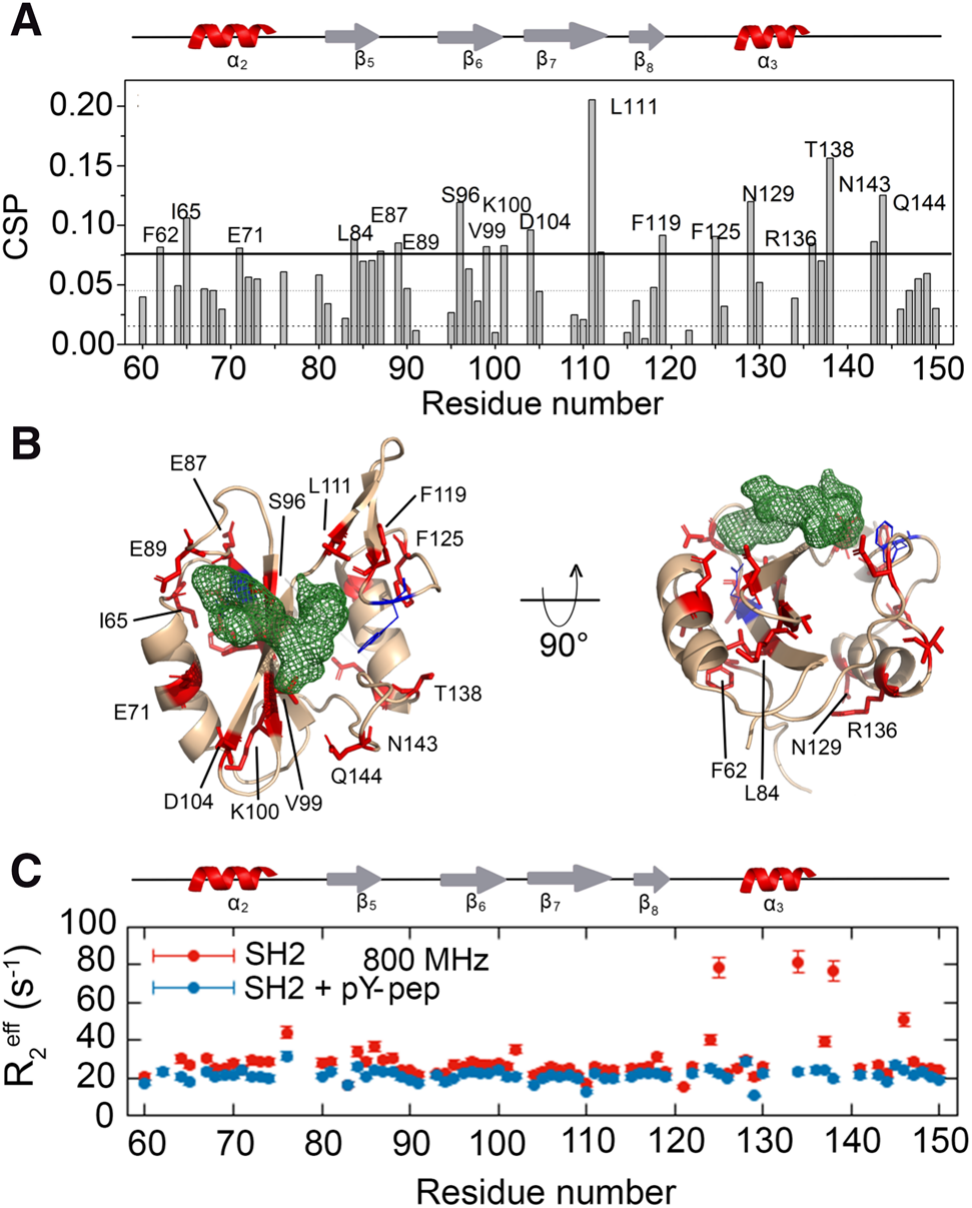
Binding of pY-pep to Grb2-SH2. (**A**) Chemical shift perturbation (CSP) as a function of residue number promoted by the presence of 2.1 mM pY-pep to Grb2-SH2 at 298 K. The labeled residues are the ones with CSP larger than the average plus two standard deviations. (**B**) Cartoon representation of the Grb2-SH2 structure (PDB_id 1BMB) highlighting in red the residues labeled in A. The green mesh is the atomic probability density map of the ensemble of all Grb2-SH2 structures complexed with phosphopeptides available at the PDB website. It presents the highest probability density of finding each atom of the phosphopeptides, which contains the pY, pY + 1, and pY + 2. (**C**) R_2*eff*_ at 66.7 s^-1^ as a function of the residue number at 18.8 T (800 MHz) for the Grb2-SH2 with (blue) and without (red) pY-pep at a saturated concentration (6 mM) at 283 K. For the complete results see Figures S4, S8, and S9.

To correlate the dynamics with the phosphotyrosine sensing mechanism, we measured the effect of pY-pep binding on the Grb2-SH2 structure and dynamics. We analyzed the chemical shift perturbation (CSP) upon pY-pep binding (Fig. 4A). There were significant perturbations throughout the protein. When we looked at the most prominent CSPs at Grb2-SH2 structure (Fig. 4B), we verified the correlation with the peptide binding. Based on all the information available on PDB for complexes between Grb2-SH2 and different phosphopeptides, we calculated the atomistic probability density for the phosphopeptides bound to Grb2-SH2. The green mesh at Fig. 4B shows that the highest probability density is at positions pY, pY + 1, and pY + 2, in agreement with the CSP. Many of the perturbed residues are directly facing the atomic probability density of the phosphopeptides (green mesh), at subdomain I, E89 in the loop β5/β6, the same regions observed in the calculated structure as open (Figure S2D), R86 that directly binds to pY, and S96. At subdomain II, L111 of the β-hairpin β7/β8 (the highest CSP) and Q144 also bind directly to the phosphopeptide. In contrast, the probability density map of the backbone of the bound structures in the PDB is closed (Figure S2D). Residue F62, N129, and R136 are on the opposite face. It is noteworthy that in the full-length Grb2 the F62 and R136 are facing the dimerization interface. It was previously reported^18^ that the binding of pY-pep leads to the dissociation of the dimeric Grb2. The binding of pY-pep also affected residues at α3 (N129 and T138). L111 and T138 were the ones with the highest CSP.

Next, we measured the ^15^N CPMG-RD for a saturated concentration of pY-pep (6 mM) (Fig. 4C, S8, and S9). At this condition, most of the conformational exchange observed for subdomains I and II is quenched. This is typical of a conformational selection mechanism of binding.

**Figure 5:**
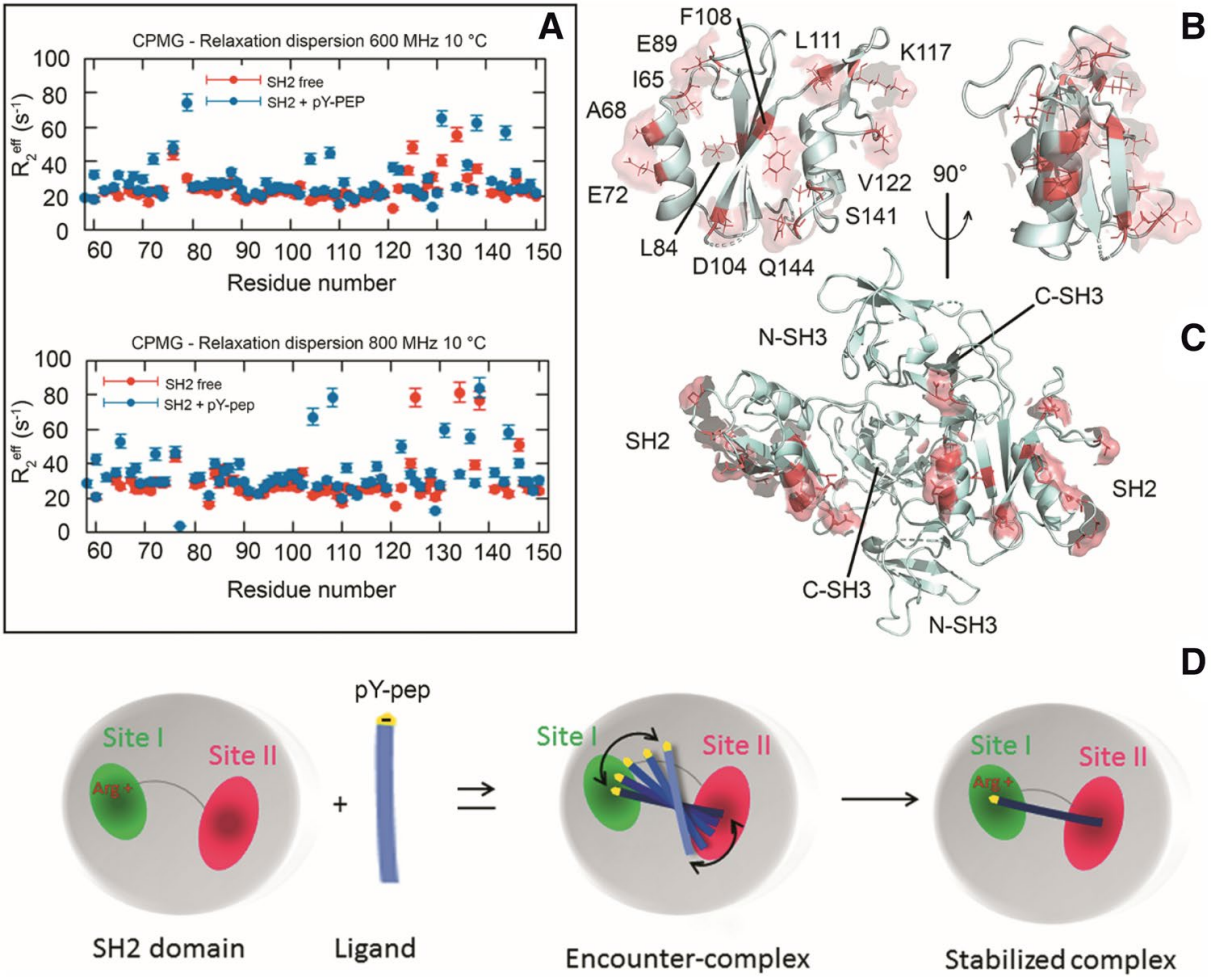
Effect of the binding of pY-pep to Grb2-SH2 domain at a semi-saturated concentration. (**A**) R_2*eff*_ at 66.7 s^-1^ as a function of the residue number in two fields, 14.09 (600 MHz) and 18.8 (800 MHz) T, for the Grb2-SH2 with (blue) and without (red) pY-pep at a semi-saturated concentration (2.1 mM) at 283 K. For the complete ^15^N CPMG-RD profiles see Figures S10 and S11. (**B**) Cartoon representation of the Grb2-SH2 structure showing in pink the residues that acquired relaxation dispersion in the presence of a semi-saturated concentration of pY-pep. Note that the residues which gained exchange are exclusively at the pY-pep molecular recognition face. (**C**) Cartoon representation of the full-length Grb2, showing in pink the residues that gained exchange. It is worth to note that they are solvent-exposed on the surface, and not involved in inter-domain or inter-subunit contacts. (**D**) Schematic representation of the proposed molecular recognition mechanism for the phosphopeptides. The pY-pep becomes tethered to the recognition surface, forming an encounter complex and finally finds the recognition site stabilizing the complex. The uncertainty of the position of pY-pep at the encounter complex induces the observed chemical exchange.

We also measured the ^15^N CPMG-RD for a semi-saturated concentration of pY-pep (2.1 mM) (Fig. 5). We took advantage of the chosen experimental conditions at 20 mM inorganic phosphate and 200 mM NaCl. The inorganic phosphate competes with the pY-pep for the binding to R86 while the NaCl shields electrostatic interactions. At this condition the pY-pep binds with millimolar affinity, enabling the observation by NMR of “binding intermediates” in the process of molecular recognition. We observed, in the presence of a semi-saturated concentration of pY-pep, that residues acquired relaxation dispersion (Fig. 5A). Interestingly, these residues are at the same surface of the recognition site as shown in Fig. 5C. It is noteworthy that the residues that took part in exchange processes are uniquely at the pY-pep molecular recognition face. In the context of the full-length Grb2, this surface is exposed to the solvent, not involved in inter-domain or inter-subunit contacts.

We proposed a mechanism for pY-pep molecular recognition that involves the formation of an encounter complex as “binding intermediates” in the process (Fig. 5D). The recognition surface of the encounter complex is wider than that of the stabilized complex. Fuzzy interactions of pY-pep led to an uncertainty of the position of the pY-pep at the encounter complex that generates a chemical exchange that could be observed by NMR. In this mechanism, the residues that gained exchange are those involved in the tethering of pY-pep (or pY-pep´s) in the encounter complex. It is interesting to observe that some of these residues are negatively charged (E72, E89, D10), which were surrounding the positively charged R86 at site I, the responsible for the direct binding to the phosphate of phosphotyrosine. Hydrophobic residues also were found in the encounter complex (I65, A68, L84, F108, L111, and V122). It is noteworthy that L111 showed the highest CSP, no conformational exchange in the free state and gained relaxation dispersion in the presence of semi-saturated concentrations of pY-pep (Figure S10 and S11). Ross & Subramanian^28^ described the importance of hydrophobic surfaces in the formation of encounter complexes, with the first step of mutual penetration of hydration layers causing solvent disorder followed by further short-range interactions.

**Figure 6:**
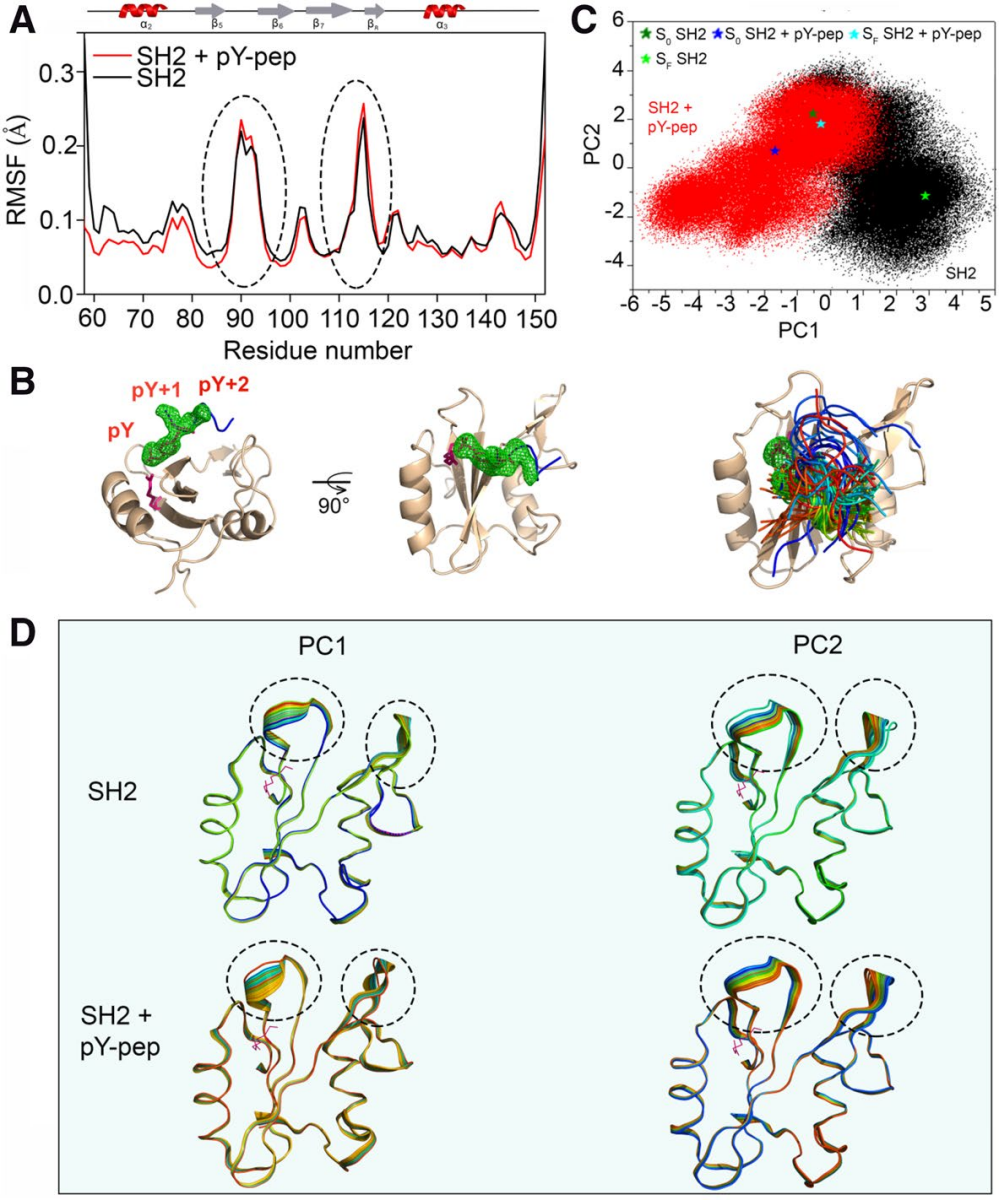
Molecular dynamic simulation of free and pY-pep bound Grb2-SH2 domain. (**A**) RMSF of Grb2-SH2 in the presence (red) and absence (black) of pY-pep. (**B**) Cartoon representation of the structure of Grb2-SH2 (PDB_id 1BMB). The green mesh is the atomistic probability density map of the ensemble of pY-pep structures along with the MD simulation. It represents the probability density of finding each atom of the phosphopeptide, which contains the pY, pY + 1, and pY + 2. Note that there is no probability density shown for the residues pY + 3, pY + 4, and pY + 5. The structure in the bottom shows the superposition of the pY-pep structure with the interaction surface of Grb2-SH2 along with the MD simulation (at each 10 ns). Note the high variability in position relative to the domain for pY + 3, pY + 4, and pY + 5. (**C**) PC1 x PC2 observed in the PCA for Grb2-SH2 in the presence (red) and absence of pY-pep (black). Note that both in the presence or in the absence of pY-pep, the MD simulation shows transitions between conformational sub-states. The colored stars show the starting points of the MD simulations (S0 dark green for the free domain and dark blue for the complexed domain) and the ending point of the MD-simulations (SF, dark green for the free domain and dark blue for the complexed domain). (**D**) Flexibility observed in the PCA for the first principal eigenvector (PC1) and second principal eigenvector (PC2) for Grb2-SH2 in the presence and absence of pY-pep. The dotted circles in A and C highlight loop β5/β6 and the hairpin β7/β8, which are the main flexible regions in the free or complexed Grb2-SH2 domain along with the MD simulation.

To better understand the dynamics of Grb2-SH2 domain and the mobility of pY-pep relative to the domain, we run 1 µs molecular dynamics simulations (MD) for the free domain and complexed with the pY-pep. By analyzing the root mean square fluctuation (RMSF, Fig. 6A), we observed four main conformational flexible regions, the N- and C-terminal residues, the loop β5/β6, and the hairpin β7/β8, both in the presence and absence of pY-pep (Fig. 6A). The conformational flexibility in these regions could not be observed in the nuclear spin relaxation parameters (Figure S2), which reflect the thermal flexibility in a timescale smaller than the overall rotational correlation time (τ_c_, sub-τ_c_ dynamics). Based on the R_2_/R_1_ ratio, we estimated τ_c_ to be ~ 6.5 ns. The motions observed in the MD simulations are probably related to supra-τ_c_ dynamics (tenths of ns to  µs), which could not be observed either by ^15^N nuclear spin relaxation parameters or ^15^N CPMG-RD. Supra-τ_c_ dynamics are described to regulate the “on” rates in the molecular recognition process^29^.

In the complex, there was a slight increase in supra-τ_c_ flexibility for loop β5/β6 and hairpin β7/β8, and the C-terminal loop between residues 140 and 150. There was also a decrease in supra-τ_c_ flexibility for residues in α2, loop α2/β5, and loop β6/β7. This observation corroborates with the CSP results observed upon the addition of pY-pep (Fig. 4A,B), for which these regions presented significant CSP values. Highlighting that L111 in the hairpin β7/β8, and N143 and Q144 in the C-terminal loop are very important residues for the binding and L111 is part of the encounter complex (Figure S10 and S11), demonstrating the importance of these regions to the recognition of pY-pep and the complex formation. This behavior of increased flexibility of Grb2-SH2 in the presence of pY-pep was corroborated by the analysis of the root mean square deviation (RMSD) relative to the starting structures (Figure S12A). In the presence of pY-pep there is a wider fluctuation of RMSD around its average value when compared with the free domain. We also observed a stability point for the values of RSMD after ~ 500 ns for the free Grb2-SH2 domain, while for the pY-pep bound domain this stability point was observed since the beginning of the simulation.

The MD simulations helped describe the position and mobility of pY-pep relative to the domain. As expected, positions pY, pY + 1, and pY + 2 were well determined, presenting a high atomistic probability density at these positions (Fig. 6B). In contrast, the positions pY + 3, pY + 4, and pY + 5 showed high mobility and low atomistic probability density. This behavior agrees with the expected specificity mapping for Grb2-SH2^30^, with no participation of pY + 3. It is remarkable that even though R86( +)/pY(-) interaction is present along with all the MD simulation, the backbone position varies significantly for all peptide, even at the postions pY and pY + 1, showing the presence of multiple conformations of the peptide at the recognition face (Fig. 6B).

To describe the supra-τ_c_ motions, we analyzed the trajectories of the MD simulations using principal component analysis (PCA). The two main principal-components (PC1 and PC2) showed an oscillation among different conformational sub-states along the simulation time (Figure S12B). For the free domain, we observed a convergence toward a stabilized conformation both in PC1 and PC2 after ~ 400 ns of simulation (2 to 3 for PC1 and 0 to − 2 for PC2 in Figure S12B). We must be careful before attributing a convergent structure for the free domain, because, with longer simulation time it could be reverted, representing a lower-frequency oscillation. For the complex, we observed no stability point, both in PC1 and PC2, without a convergence, agreeing with the gained supra-τ_c_ flexibility that denotes a higher frequency oscillation of possible conformational sub-states of the protein. The plot PC2 versus PC1 (Fig. 6C) showed the interconversion among different conformational sub-states. For both free and complexed Grb2-SH2 domain, we observed different interconverting conformational sub-states. Interestingly, the conformational sub-state with PC1 in the range between −1.5 and 1 and with PC2 between 0 and 3 are common between the free and complexed domain. It is important to note that this sub-state is the starting point for the MD simulation of Grb2-SH2 and the ending point for the complexed protein. It is worth mentioning that the starting point was the crystal structure from PDB_id 1BMB^31^ striped of the complexed peptide. The PCA was done with the two MD simulations concatenated in the same trajectory file, which enabled the direct comparison of the eigenvectors PC1 and PC2 for both free and complexed MD simulations.

The structural extent of supra-τ_c_ conformational dynamics is depicted in Fig. 6D. Note that loop β5/β6 and the hairpin β7/β8 presented the main structural changes responsible for PC1 and PC2. Those are the regions with the highest RMSF and higher flexibility in the presence of pY-pep. The extent of structural change in the presence of pY-pep was more pronounced than for the free domain. This could also be observed for the C-terminal loop, containing the residues N143 and Q144. Remarkably, these are the same regions for which we observed the open-to-close conformational transition in the calculated structure (Figure S2C).

## Discussion

Here we present the dynamical behavior of Grb2-SH2 domain and its relationship with the molecular recognition of phosphopeptides. We showed that this domain behaves as two dynamically independent subdomains, suggesting a correlation with previously described properties of Grb2-SH2. There are many reports on the dynamics of SH2 domains. However, a full characterization of an SH2 domain in the milli-to-microsecond timescale was not available. Many of the contributions correlate backbone dynamics in the pico-to-nanosecond timescale with the binding of phosphopeptide recognition^32–37^. In the present manuscript we described the dynamical behavior in multiple timescales, but with an emphasis on the conformational exchange (milli-to-microseconds), which is the timescale related to molecular recognition (k_off_).

The dynamical data showed that subdomain I is involved in microsecond dynamics while subdomain II in millisecond dynamics, suggesting independence in the association of the pY recognition (subdomain I) and pY + 2 recognition (subdomain II). We proposed that the dynamics at subdomain II involves a minor conformational change that exposes hydrophobic residues to the solvent. We present here the first dynamical characterization of Grb2-SH2, revealing a torsion/reorientation mechanism of α3, an important conformational state for the binding specificity of pY + 2 and allosteric events.

Our data showed that pY-pep recognition occurred by conformational selection, in which a conformational state is stabilized. The phosphopeptide association is specific for the exposed-face of the full-length Grb2, the recognition-face (Fig. 5C). Before the formation of a stabilized-complex, an encounter complex was formed involving negatively charged and hydrophobic residues, creating an electrostatic potential that drives the association of the phosphotyrosine (pY) to R86 (Figure S14A). This fact suggests that the association can be driven by Coulombic and hydrophobic interactions. The observation of an encounter complex led to the conclusion that the recognition face is wide, with fuzzy interactions with the phosphopeptide. There are still many unanswered questions regarding the phosphopeptide recognition by SH2 domain. One of the most intriguing is regarding the recognition of remote phosphotyrosines^38^. This is biologically relevant for proteins with multiple phosphorylations. This manuscript did not fully explain this effect, but the wide recognition interface and the fuzzy interaction with the phosphopeptide point toward a possible mechanism of how remote phosphotyrosines are sensed by SH2 domain.

The high-resolution structure of the free-state (PBD_id 6VK2) enabled the characterization of an open conformation, in which β5/β6 and C-terminal loops undergo an open-to-close conformational transition upon binding of phosphopeptides. These loops, along with the hairpin β7/β8, are far apart, demonstrating that the recognition surface is wide. The structural changes are in agreement with CSP and dynamic description of the free and bound-states, and the presence of “binding intermediates” in the encounter complex.

The PCA of the MD simulations showed that there are different conformational sub-states for the free and complexed domain, where the pY-pep can assume multiple conformations since the position pY presented the highest atomistic probability density, followed by pY + 1 and pY + 2, and the conformations at positions pY + 3 to pY + 5 were very diverse. Interestingly, the presence of pY-pep increased the extent of conformational transitions among the different sub-states. In agreement with our experimental results, Lindfors et al. (2012)^39^ used spin-labeled phosphopeptides to probe the interaction with SH2. The peptide assumes multiple orientations at the interacting surface. The electrostatic interactions involving charged patches of the protein resulted in an ensemble of rapidly exchanging orientations, suggesting a dynamic encounter state. This behavior shows that high-affinity binding can be dynamic and that residues outside the central SH2 binding site are also important for the Src-FAK interaction^39^.

The formation of the dynamic association and encounter complexes is a millisecond timescale event and involved the quenching of the dynamics of subdomain I and subdomain II. The MD simulations pinpointed important motions probably in a faster supra-τ_c_ dynamics, which involves mainly three regions of the protein: loop β5/β6 at subdomain I, and hairpin β7/β8 and the C-terminal loop, both at subdomain II. Multiple timescale dynamics are pivotal for recognition events. It is noteworthy that the slow µs-ms dynamics is related to the “off” rates, while the supra-τc dynamics (tenths of ns to µs) to the “on” rates in a molecular recognition process^29^. The wide and dynamic character of the recognition face is also evidenced by the MD simulation. There is a good correspondence between the contact map of Grb2-SH2 (Figure S13) and pY-pep and the CSP. All the regions mapped by CSP and CPMG relaxation dispersion were observed in the MD-simulation.

Our data also showed that the association of pY-pep to the recognition-face led to chemical shift changes in the opposite face, such as F62 and R136 that are involved in dimeric contacts in the full-length Grb2. The F62 is at subdomain I and R136 at subdomain II. This observation is suggestive of an allosteric event triggered by pY-pep association, which is in agreement with the fact that the association of pY-pep leads to the monomerization of full-length Grb2^18^. The monomer–dimer equilibrium of Grb2 is biologically relevant and pivotal for the regulation of signal transduction pathways ^3–6^. The Grb2 dimer interacts with the fibroblast growth factor receptor 2 (FGFR2) which in turn activates the Ras/MAPK pathway, while its monomeric form is inhibitory for the signaling. Ras/MAPK is important and widely studied via related to a variety of cancer and development defects^2,3^. Further studies are necessary for a better understanding of the mechanism of monomerization. Plasticity is a major feature of an adaptor protein such as Grb2. The present manuscript detailed many of the residues involved in the dynamic of the free and bound states of the Grb2-SH2 domain, which is the basis for future studies of the plasticity of Grb2.

The dynamics of subdomain II, showed the exposure of hydrophobic surfaces with the repositioning/reorientation of α3. The possibility of this dynamics to contribute to the domain swap was considered, once the α3 is the helix involved in the swapping mechanism^13–16^. However, our results showed that the observed dynamics is not involved in the swapping mechanism. Domain swap for Grb2-SH2 domain occurs in a different timescale (minutes/hours) from the observed α3 dynamics (ms) and the swapped dimer can be separated in a gel-filtration column. Moreover, we showed that Grb2-SH2 is monomeric in the major and minor states. The biological role of the domain swap in the Grb2-SH2 is not fully understood and further studies are necessary. Grb2-SH2 domain-swap would add another degree of freedom for Grb2 plasticity and could be important, but it was never observed in the full-length Grb2.

In conclusion, we propose a mechanism of phosphotyrosine recognition by Grb2-SH2 domain, which involves a wide recognition face and a dynamic association of the phosphopeptide. We verified the formation of an encounter complex as “binding intermediates” involving the exposed face of the SH2 domain of the full-length Grb2. This is biologically relevant for an adaptor protein, such as Grb2, since it potentially increases the scope of interaction. In agreement with this conclusion, it has been reported that Grb2 is able to sense remote phosphotyrosines. Huang et cols (2017) showed that the binding affinity for the linker of activation of T-cells (LAT) and Grb2 depends on the phosphorylation of remote tyrosine sites^38^. This capacity could be conferred by the dimeric stated of full-length Grb2, but also by the wide and dynamic character of SH2 domain to recognize phosphopeptides.

## Materials and methods

### Protein expression and purification

The SH2 domain – 6 × histidine tagged (Grb2-SH2)—was expressed and purified as previously described by Sanches et al. (2019)^19^. Chemical shifts are deposited in the Biomagnetic Resonance Bank (www.bmrb.wisc.edu) under accession number 27781.

### NMR spectroscopy

All NMR samples of Grb2-SH2 was in 20 mM sodium phosphate buffer, 200 mM NaCl, pH 7.0, 10% D_2_O. NMR spectra were acquired at 25 ºC on Bruker Avance III 600 MHz, Avance III 800 MHz and Avance IIIHD 900 MHz, equipped with ^15^N/^13^C/^1^H triple-resonance probes (Bruker TXI). NMR spectra were processed with NMRPipe^40^ and analyzed using CCPNmr Analysis software^41^. The detailed experimental setup for all NMR experiments are in Table S3.

### Structure calculation

Distance restrains were derived from ^15^N-NOESY-HSQC and aliphatic and aromatic ^13^C-NOESY-HSQC collected at an AVANCE III HD 900 MHz, for aromatics and aliphatics. The 3D-NOESY-HSQCs were obtained using non-uniform sampling (NUS, 50%) with multidimensional Poisson Gap scheduling. NMRPipe and iterative soft threshold (hmsIST) fast reconstruction of NMR data were used for processing^42^ (Table S3). We run TALOS-N^43^ for backbone chemical shift-based dihedral angle prediction. The predicted backbone dihedral angles ϕ and ψ of the residues involved in secondary structure were used as a restraint for structural calculations.

Structure calculation of the Grb2-SH2 was performed iteratively using ARIA 2.1 program, version 2.3^44,45^ combined with CNS version 1.2^46^, using ^15^N-NOESY-HSQC and aliphatic and aromatic ^13^C-NOESY-HSQCs datasets as the source of distance restraints. The CCPN/Aria interface was used^41^.

The converged structure using Aria/CNS generated an ensemble of well-converged structures (Figure S1A) that was generated from 697 unambiguous and 277 ambiguous distance restraints. Next, the structural calculation proceeded using Chemical-Shift Rosetta (CS-Rosetta, ROSETTACOMMON Version 3.7 run at NMRBOX^47^. All the intra-residue NOEs were removed in CS-Rosetta calculation. The sequential, medium, and long-range distance restraints generated using Aria/CNS were then converted to the Rosetta format and used as distance restraints. ^13^C, ^15^N, and ^1^H NMR chemical shifts were used as inputs (BMRB 27781^19^ for fragment picking, along with 479 NOE-based distance restraints. 66,317 structures generated by the standard CS-ROSETTA protocol. The 20 lowest energy structures were deposited in the protein data bank (PDB id 6VK2).

The structural ensemble was visualized and analyzed with PyMOL. Quality validation was Protein Structure Validation Software suite (PSVS) (https://montelionelab.chem.rpi.edu/PSVS/) and Molprobity (https://molprobity.biochem.duke.edu/)49.

### Nuclear spin relaxation parameters

^15^N backbone amide relaxation parameters (^15^N R_1_, ^15^N R_2_ and ^1^H-^15^N heteronuclear Nuclear Overhauser effect—NOE) were measured for a ^15^N labeled Grb2-SH2 (300 µM, in 20 mM sodium phosphate buffer, 200 mM NaCl, 10% D_2_O, pH 7.0) using Avance III HD 800 (18.8 T, operating at 800.4 MHz) at 25 °C. R_1_ was measured with delays ranging from 0.05 to 1 s. R_2_ was measured with delays varying from 17 to 170 ms. The experimental error was evaluated from the signal-to-noise ratio of the spectra. The ^1^H-^15^N NOE was acquired with or without proton saturation for 6.0 s. Details of the NMR experiments are in Table S3. The R_1_ and R_2_ values were obtained using the relaxation module of CcpNmr Analysis ^41^. The ^1^H-^15^N heteronuclear NOE values were determined using the intensity saturation spectra/intensity without the saturation spectra ratio.

### ^15^N Relaxation dispersion measurements

^15^N Carr–Purcell–Meiboom–Gill relaxation dispersion (CPMG-RD) profiles for a ^15^N labeled Grb2-SH2 (300 µM, in 20 mM sodium phosphate buffer, 200 mM NaCl, 10% D_2_O, pH 7.0) were recorded in Bruker Avance III 600 (14.09 T, operating at 600.03 MHz) and Avance III 800 (18.8 T, operating at 800.4 MHz) at four temperatures (278, 283, 290 and 298 K) using the constant relaxation time of T_relax_ = 30 ms (^15^N CPMG relaxation compensation^49^. R_2_^*eff*^(ν_CPMG_) were calculated from peak intensities (I) in a series of two-dimensional (2D) ^1^H-^15^N correlation spectra recorded in an interleaved way at different CPMG frequencies ν_CPMG_, (ranging from 66.7 to 1,000 s^-1^) using the following equation: $$R_{2}^{{eff}} \left( {\nu _{{CPMG}} } \right) = ~ - {\raise0.7ex\hbox{$1$} \!\mathord{\left/ {\vphantom {1 {T_{{relax}} }}}\right.\kern-\nulldelimiterspace} \!\lower0.7ex\hbox{${T_{{relax}} }$}}\ln \left( {{\raise0.7ex\hbox{$I$} \!\mathord{\left/ {\vphantom {I {I_{o} }}}\right.\kern-\nulldelimiterspace} \!\lower0.7ex\hbox{${I_{o} }$}}} \right)$$, where I is the signal intensity in the spectra collected at T_relax_ = 30 ms and I_0_ is the signal intensity in the reference spectrum recorded at T_relax_ = 0. The experimental error in R_2eff_ rates were estimated signal to noise ratio for each resonance $$\Delta R_{2}^{{eff}} \left( {\nu _{{CPMG}} } \right) = ~{\raise0.7ex\hbox{$1$} \!\mathord{\left/ {\vphantom {1 {T_{{relax}} }}}\right.\kern-\nulldelimiterspace} \!\lower0.7ex\hbox{${T_{{relax}} }$}}\left( {{\raise0.7ex\hbox{$1$} \!\mathord{\left/ {\vphantom {1 {(SIGNAL/NOISE)}}}\right.\kern-\nulldelimiterspace} \!\lower0.7ex\hbox{${(SIGNAL/NOISE)}$}}} \right)$$.

### Amide chemical shift temperature coefficient

Amide ^1^HN chemical shift temperature coefficient of Grb2-SH2 (300 µM, in 20 mM sodium phosphate buffer, 200 mM NaCl, 10% D_2_O, pH 7.0) was measured in the presence and absence of the pY-pep. A series of two-dimensional ^15^N/^1^H HSQC spectra at 278, 283, 290 and 298 K was acquired (Bruker Avance III HD 800 18.8 T, operating at 800.4 MHz). The water chemical shift was referenced using 3-(trimethylsilyl)propane-1-sulfonic acid (DSS). Each spectrum was referenced to the water signal at each temperature. They were processed using NMRPipe and analyzed using CcpNmr Analysis. The amide chemical shift values (δ_HN_) of all residues at different temperatures were plotted as function of the temperature, and the slope (dδ_HN_/dT) of every curve was plotted for each Grb2-SH2 residue.

### Phosphopeptide binding

The binding of the phosphopeptide EpYINSQV (sequence from the Epidermal Growth Factor receptor, pY-pep^18^ to Grb2-SH2 (300 µM, in 20 mM sodium phosphate buffer, 200 mM NaCl, 10% D_2_O, pH 7.0, 298 K) was evaluated by chemical shift perturbation (CSP) using the ^15^N-HSQC spectra with and without pY-pep. CSP values were calculated using the equation $CSP=\sqrt{{0.5\left({\Delta \delta }_{H}\right)}^{2}+{0.5\left({\Delta \delta }_{N}/10\right)}^{2}}$.

### Construction of the probability map

We created a map of the weighted atomic density using the function VOLMAP in Visual Molecular Dynamics (VMD)^50^ software. The atomistic probability map was calculated (i) over an ensemble containing all Grb2-SH2 complexed with phosphopeptides available at the protein data bank (RSCB PDB), except the domain-swapped Grb2-SH2 dimers and (ii) an ensemble of 100 structures collected at each 10.000 frames of the molecular dynamics simulation of Gbr2-SH2 and of Grb2-SH2 complexed with the docked pY-pep.

### Computational simulations of the free and pY-pep bound Grb2-SH2 domain

The structure of the Grb2-SH2 domain used for computer simulations was downloaded from PDB website under access code 1BMB^31^. The phosphopeptide and water molecules in the crystal structure were removed. The domain and pY-pep were prepared using AutoDockTools program^51^ for molecular docking simulations, merging non-polar hydrogen atoms and adding atom types. The pY-pep rigid root was generated automatically, setting all possible rotatable bonds defined as active by torsions. The protonation states of ionizable residues of the protein were defined according to PROPKA software^52^ from PDB2PQR webserver^53^, considering pH 7.0. The molecular docking calculations were performed by using AutoDock Vina^54^, applying a total of 16 exhaustiveness. The coordinates of the center of the conformational search box at the protein binding site were defined according to the CSP results and the atomic probability density map generated from Grb2-SH2 crystal structures complexed with phosphopeptides available in PDB website. The box dimensions were 30 × 26 × 26 Å on the three coordinate axes.

Molecular dynamics (MD) simulations were performed in GROMACS version 5.0.1^55^ using the AMBER99SB-IDLN force field^56^ for modeling the Grb2-SH2 domain and pY-pep. TIP3P^57^ was used as water model. The starting position of the pY-pep for the MD simulations was obtained from the molecular docking calculations. The structures of the free protein (PDB-ID 1BMB) and complexed with pY-pep (from AutoDock Vina) were each placed in the center of a 66 Å cubic box solvated by a solution of 200 mM NaCl in water, and the protonation state of ionizable residues was determined by PROPKA results, considering a pH 7.0. The periodic boundary conditions and NPT ensemble were used in all simulations, keeping the systems at 298 K and 1.0 bar using Nose–Hoover thermostat ($\tau_{T}$ = 2.0 ps) and Parrinello-Rahman barostat ($\tau_{P}$ = 2.0 ps and compressibility = 4.5 × 10^–5^ bar^-1^). A 12 Å cut-off point for the Lennard–Jones and Coulomb potentials was used. The long-range electrostatic interactions were calculated using the particle mesh Ewald (PME) algorithm. The simulations were performed using a time step of 2.0 fs and all covalent bonds involving hydrogen atoms were constrained to their equilibrium distance. A conjugate gradient minimization algorithm was utilized to relax the superposition of atoms generated in the box construction process. Energy minimizations were carried out with steepest descent integrator and conjugate gradient algorithm, using 500 kJ mol^-1^ nm^-1^ as maximum force criterion. At the end of preparation, 1.0 μs MD simulation of the free and pY-pep bound Grb2-SH2 were performed for data acquisition. Following dynamics, the trajectories of free and pY-pep bound domain were firstly concatenated individually and analyzed according to root mean square deviation (RMSD) of backbone atoms and root mean square fluctuation (RMSF) with respect of the C_□_ atoms of the protein. A total of 100 equally spaced frames over MD simulation time containing only pY-pep structural information were extracted for the evaluation of mobility of the phosphopeptide and for the also construction of the atomic probability density map as mentioned in the previous section. After individual analysis of each simulation, the trajectories for the free and pY-pep bound protein excluding the initial 1.0 ns were concatenated in a single file, and this new trajectory was used to the Principal Component Analysis (PCA). This aspect is important because ensures the same eigenvectors (PC1 and PC2) for both MD simulations, enabling us to directly compare the occurrence of conformational sub-states. The average structure used as a reference in the PCA calculations was obtained from the first 9.0 ns. The values of PC1 and PC2 were calculated considering the respective portions of simulation time for the free and complexed protein. For a visual inspection of motions related to the principal components, 30 frames containing Grbs2-SH2 structural information were extracted from PC1 and PC2 in the simulation time ranges correspondent to the free and pY-pep bound protein. The structural representations were prepared using PyMol^58^.

## Acession numbers

The atomic coordinates, experimental restraints and chemical shift assignments are available at the Protein Data Bank (PDBID 6VK2) and Biomagnetic Resonance Data Bank (BMRB ID 27781).

## Supplementary information

Supplementary information

## Abbreviations

Grb2: Growth factor receptor-bound protein 2
SH2: Src homology 2
MAPK: Mitogen activated protein kinase
NMR: Nuclear magnetic resonance
CPMG: Carr–Purcell–Meiboom–Gill pulse train
CSP: Chemical shift perturbation
RMSD: Root mean square deviation
RMSF: Root mean square fluctuation
