# Supplementary material for: The dynamics of free and phosphopeptide-bound Grb2-SH2 reveals two dynamically independent subdomains and an encounter complex with fuzzy interactions
Source: Sci Rep. 2020 Aug 3;10:13040. doi: 10.1038/s41598-020-70034-w (PMC7398917; doi:10.1038/s41598-020-70034-w)
Supplement: Supplementary file 1 — Supplementary information [file 41598_2020_70034_MOESM1_ESM.docx]

**Supplementary Material**

**The dynamics of free and phosphopeptide-bound Grb2-SH2 reveals two dynamically independent subdomains and an encounter complex with fuzzy interactions.**

Karoline Sanches§, Icaro P. Caruso€‡§, Fabio C. L. Almeida€‡*, Fernando A. Melo§*

€Institute of Medical Biochemistry – IBqM, Federal University of Rio de Janeiro, Rio de Janeiro, Brazil.

‡National Center for Structural Biology and Bioimaging (CENABIO) / National Center for Nuclear Magnetic Resonance (CNRMN), Federal University of Rio de Janeiro, Rio de Janeiro, Brazil.

§Multiuser Center for Biomolecular Innovation (CMIB), Department of Physics, Sao Paulo State University (UNESP), Sao Jose do Rio Preto, Sao Paulo, Brazil

*Correspondence to: F.C.L. Almeida, falmeida@bioqmed.ufrj.br

F. A. Melo, fernando.melo@unesp.br

**DESCRIPTION OF THE STRUCTURE CALCULATION**

We assigned ^1^ and calculated the structure of the Grb2-SH2 domain at pH 7.0. Here, we present a solution structure of the free state of Grb2-SH2 domain with improved resolution, the first at pH 7.0. We used a combination of Aria2/CNS and chemical shift Rosetta (CS-Rosetta) calculations. The first round of structures was calculated using Aria2/CNS with iterative NOE assignments, which resulted in a reasonably well-converged ensemble of structures (Figure S2A). The molecular fragment replacement strategy implemented by Rosetta is a robust way to calculate accurate and high-quality solution structures when a scarce source of experimental restraints is available^2^, especially when NOE-derived distance restraints are used. We calculated the final structural ensemble using CS-Rosetta^3^ with the ambiguously and unambiguously assigned NOEs derived from Aria/CNS (first round) (Figure S2B and S2C, Table S2). There was an improvement in the convergence and geometric quality, with better positioning of the loops (Table S2). The structural ensemble (PDB_id 6VK2) presented a good consistency with the experimental NOEs. The protein folding and positioning of the loops and secondary structure elements were highly driven by the NOEs (total of 479 NOEs, being 191 long-range). Figure S2C shows the convergence of CS-Rosetta calculations.

The high resolution of the loops enabled the comparison of the structural ensemble with the phosphopeptide-bound conformations, represented by the atomistic probability density map constructed from all available Grb2-SH2 bound-structures (Figure S2D). The main differences between the bound and free ensembles are in the loop β5/β6, hairpin β7/β8, and C-terminal loop (loop between residues 140 and 150) (Figure S2D). Remarkably, the β5/β6 and C-terminal loops are opened in the free structure and closing upon the binding to the phosphopeptide. Further on, we will show that these regions undergo changes in dynamics, CSP, and in the MD simulation upon phosphopeptide binding. The comparison of the present structural ensemble with the available structures free in solution (1GHU and 1FHS, not shown)^4,5^ confirmed that β5/β6 and C-terminal loops are in the open conformation.

To get more insights into the structure of Grb2-SH2 in solution, we acquired ^15^N-HSQC spectra at several temperatures and calculated the HN chemical shift temperature susceptibility (dδ_HN_/dT, Figure S2E) ^6^. The dδ_HN_/dT reports on the thermal susceptibility of the amide hydrogen bonds H_N_-C´. An empirical simple rule stands that dδ_HN_/dT > -5 ppb/K for HNs involved in secondary structure^7^ (low thermal susceptibility), and HNs of residues bonded to water present high thermal susceptibility (dδ_HN_/dT < -5 ppb/K). Residues with low amide chemical shift thermal susceptibility (dδ_HN_/dT > -5 ppb/K) make less expandable hydrogen bonds, which are stronger, presenting larger hydrogen bond J coupling ^3h^J_NC´_^8^. For this, an amide with dδ_HN_/dT > -5 ppb/K is frequently involved in hydrogen bonds of secondary structures. This measurement was particularly important to independently confirm the presence of the hairpin β7/β8, which is evident in both Aria/CNS and CS-Rosetta calculation and displays dδ_HN_/dT > -5 ppb/K. This hairpin is absent in the solution structures of free Grb2-SH2 previously reported^4,5^. The values of dδ_HN_/dT for residues in β8 are significantly decreased in the presence of the pY-pep. The participation of this hairpin in the binding will be further discussed.

***Fitting of Temperature-Dependent ^15^N CPMG-RD Data***

The ^15^N CPMG-RD profiles of Grb2-SH2 were recorded at 278, 283, 290, and 298 K and two magnetic fields, 600 and 800 MHz. We fit the 15N CPMG-RD data to the Bloch-McConnel equation, which describe the evolution of the magnetization under chemical or conformational exchange. The fitting goes from the simplest two-state model to a more complex if necessary. A rigorous statistical method is necessary to evaluate the generated global model. To obtain the kinetic and thermodynamic parameters of the minor state, the data were fit together using a model of two states exchange, major state (A) and a minor-populated state (B) by minimization of the following χ^2^(ζ) target function:

$$\chi^{2}\left( \zeta\right)=\sum\frac{{({R_{2,calc}}^{eff}\left( \zeta\right)- {R_{2}}^{eff})}^{2}}{{(\Delta{R_{2}}^{eff})}^{2}} (1)$$

where R_2,calc_^eff^ is experimental relaxation rates and ΔR_2_^eff^ the experimental error. R_2,calc_^eff^ (ζ) are calculated relaxation rates obtained by numerical solution of Bloch-McConnell equations^9^. ζ={χ_1_,…,χ_npar_} is a set of adjustable model parameters, n_par_ is the number of adjustable parameters. Summation in eq. 1 is over the number of experimental data points n_dat_.

We performed the data fitting through software cpmg_fit^10,11^ assuming that all residues in Grb2-SH2 are involved in the same global exchange process, the intrinsic relaxation rates R_2_ at time zero are the same in states A and B, the chemical shift differences between states, *Δϖ_BA_*, are independent of temperature and the populations of the exchanging states and rates of transitions between states follow Boltzmann distribution (van´t Hoff) and Eyring equation, as illustrate in Figure 2C:

*p_B_*/*p_A_* = exp(-Δ*G_BA_*/(*RT*)) (2)

$$k_{B\longrightarrow A}=\frac{k_{B}\kappa T}{\hbar}e^{\left( -\frac{\Delta{G^{\dagger}}_{\mathrm{BA}}}{\mathrm{RT}} \right)} (3)$$

where Δ*G_BA_* = Δ*H_BA_*-*T*Δ*S_BA_* is free energy difference between states B and A, Δ*G^†^_BA_* = Δ*H^†^_BA_*-*T*Δ*S^†^_BA_* is activation free energy, Δ*H_BA_*, Δ*S_BA_*, Δ*H^†^_BA_* and Δ*S^†^_BA_* are equilibrium and activation entropies and enthalpies, respectively, *h, k_B_*, and, *R* are Planck’s, Boltzmann’s and the universal gas constants, respectively, and κ is a transmission coefficient (κ = 1.6·10^-7^ was used suggested for protein folding process^12–14^. The set of adjustable model parameters in the global 2-state exchange model included ∆G, Δ*H*, Δ*S*, ∆G*^†^*, Δ*H^†^* and Δ*S^†^*, chemical shift differences between states for all residues *Δϖ*, and intrinsic relaxation rates R_2_ for all residues, temperatures and magnetic fields.

To extract the population of the minor state, *p_B_*, exchange rate constant, *k_ex_* = *k_A→B_*+*k_B→A_*, *Δϖ_BA_* values for all residues and R_2_ values for all residues and magnetic fields we also performed the ^15^N CPMG-RD data analysis for the same set of residues recorded at 2 magnetic independent of each temperature. The equilibrium and activation parameters, Δ*H_BA_*, Δ*S_AB_* Δ*H^†^_BA_* and Δ*S^†^_BA_*, were then obtained from van’t Hoff linear fits of ln(*p_B_*/*p_A_*) *vs.* 1/T and ln(*k_B →A_*) *vs.* 1/T (see eqs. 2,3). The parameters obtained can be found in Table S2.

The quality of data fits was assessed by comparing the resulting values of χ^2^(ζ) target function to the number of degrees of freedom of the model DF=n_dat_-n_par_ (Table S2). The global fits of ^15^N CPMG RD data should result in χ^2^ of the order of DF and the model adequately fits the data. The values of χ^2^ target functions obtained in global fits of CPMG RD data are shown in Table S2 for the individual and global fittings. χ^2^ of the order or less than DF were obtained in all data fits, showing that the model of global two-state conformational exchange is within experimental uncertainties.


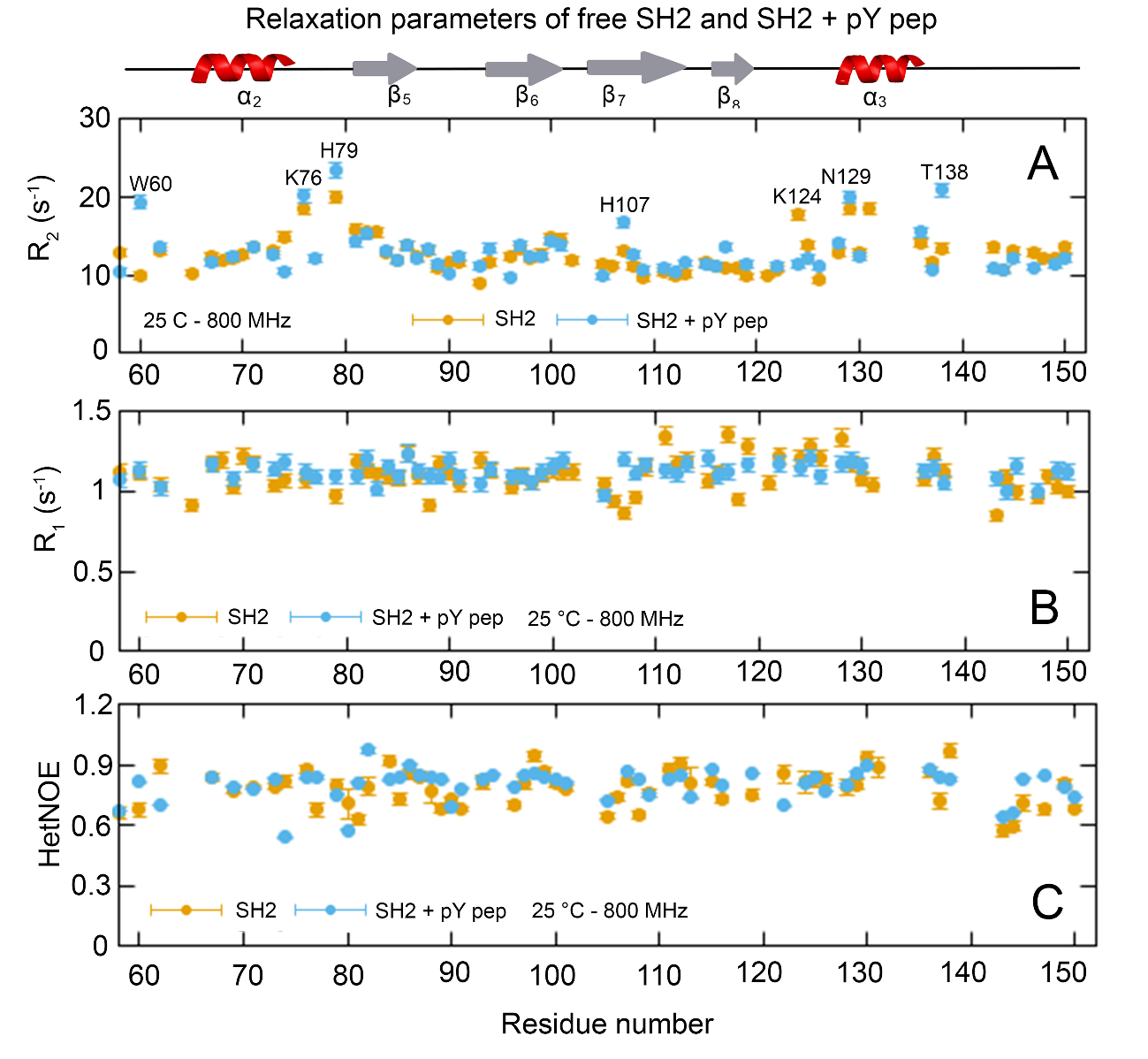


Figure S1: ^15^N relaxation parameters (R_1_, R_2_, ^1^H-^15^N heteronuclear NOE) of Grb2-SH2 domain at pH 7.0 in the presence (blue) and absence (orange) of the phosphopeptide (pY-pep).


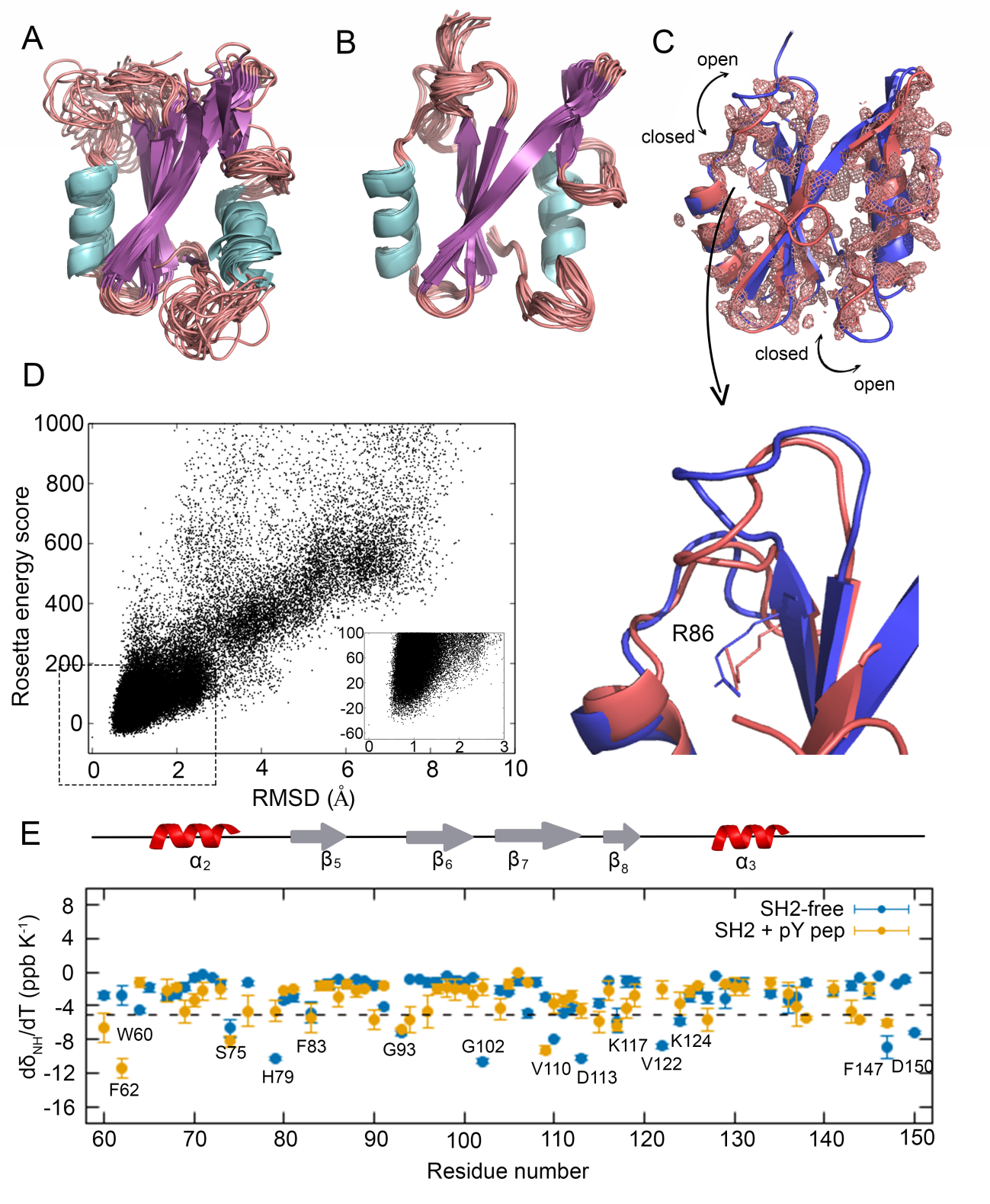


Figure S2: Structure of Grb2-SH2 domain at pH 7. (A) Ensemble of the 20 lowest energy structures calculated by Aria2/CNS. (B) Ensemble of the 20 lowest energy structures calculated by molecular fragment replacement (CS-Rosetta) and the 479 distance restraints obtained from the Aria2/CNS calculation (Table S2). (C) Superposition of the lowest energy structure (free, PDB_id.: 6VK2, blue) and the phosphopeptide bound structure (PDB_id.: 1BMB, pink). The pink mesh represents the atomic probability density map of the ensemble of all Grb2-SH2 structures complexed with phosphopeptides available at the PDB website. It presents the highest probability density of finding each atom of the Grb2-SH2 domain. The arrows show the most pronounced differences observed between the free and bound structures. The α2/β5 and C-terminal loops are opened in the free structure, and closing upon the binding to the phosphopeptide. The zoom at the bottom shows the opened-closed differences and the reorientation of the R86 side chain upon the phosphopeptide binding. (D) Energy funnel showing the convergence of the CS-Rosetta. The insert is a zoom of the bottom of the funnel. (E) H_N_ chemical shift temperature coefficient obtained for the Grb2-SH2 in the presence (orange) and absence (blue) of the phosphopeptide pY-pep.


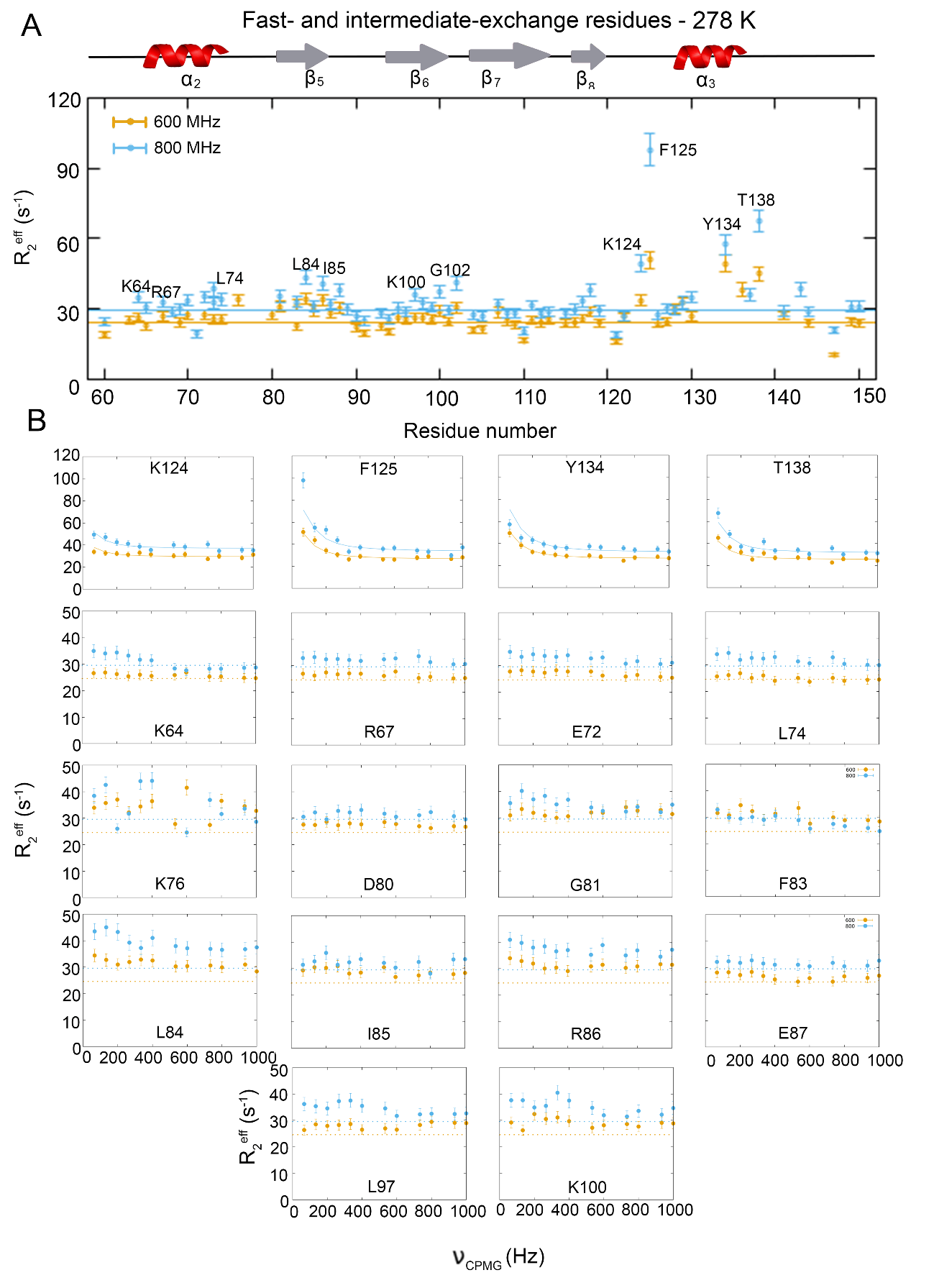


Figure S3: ^15^N CPMG relaxation dispersion profiles (R_2_*^eff^* x ν_CPMG_) at 278 K (5ºC), pH 7.0. (A) R_2_*^eff^* at 66.7 s^-1^ as a function of the residue number in two fields, 14.09 (600 MHz) and 18.8 T (800 MHz). Different dynamic regions of the protein can be distinguished based on the relaxation: (i) subdomain I: residues in fast conformational exchange (Figure 2A) and (ii) subdomain II: residues in intermediate conformational exchange. The lines show the most likely R_2_*^eff^* without exchange contribution for each dynamic region (R_2_*^eff^*∞). It is used as a reference to determine residues clearly involved in conformational exchange (labelled). The typical ^15^N CPMG-RD profile observed fast exchange regimes is where all the points are above the line, meaning that much larger values of ν_CPMG_ would be necessary to reach R_2_*^eff^*∞. The intermediate exchange regime is characterized by the dispersion curve tending to R_2_*^eff^*∞. The ν_CPMG_ is enough to refocus the conformational exchange and to reach R_2_*^eff^*∞. (B) Selected relaxation dispersion profiles of residues in conformational exchange. K124, F125, Y134, and T138 are in intermediate exchange and found at subdomain II. We also had a good relaxation dispersion profile for R136 (intermediate exchange) at the 600 MHz. The resonance of R136 at the 800 MHz was not used because it was overlapped with a folded side-chain peak. K64, R67, E72, L74, K76, D80, G81, F83, L84, I85, R86, E87, L97, and K100 are in fast exchange, found at subdomain II. The orange symbols correspond to the relaxation dispersion obtained at 14.09 T and the blue symbols to the values at 18.8 T.


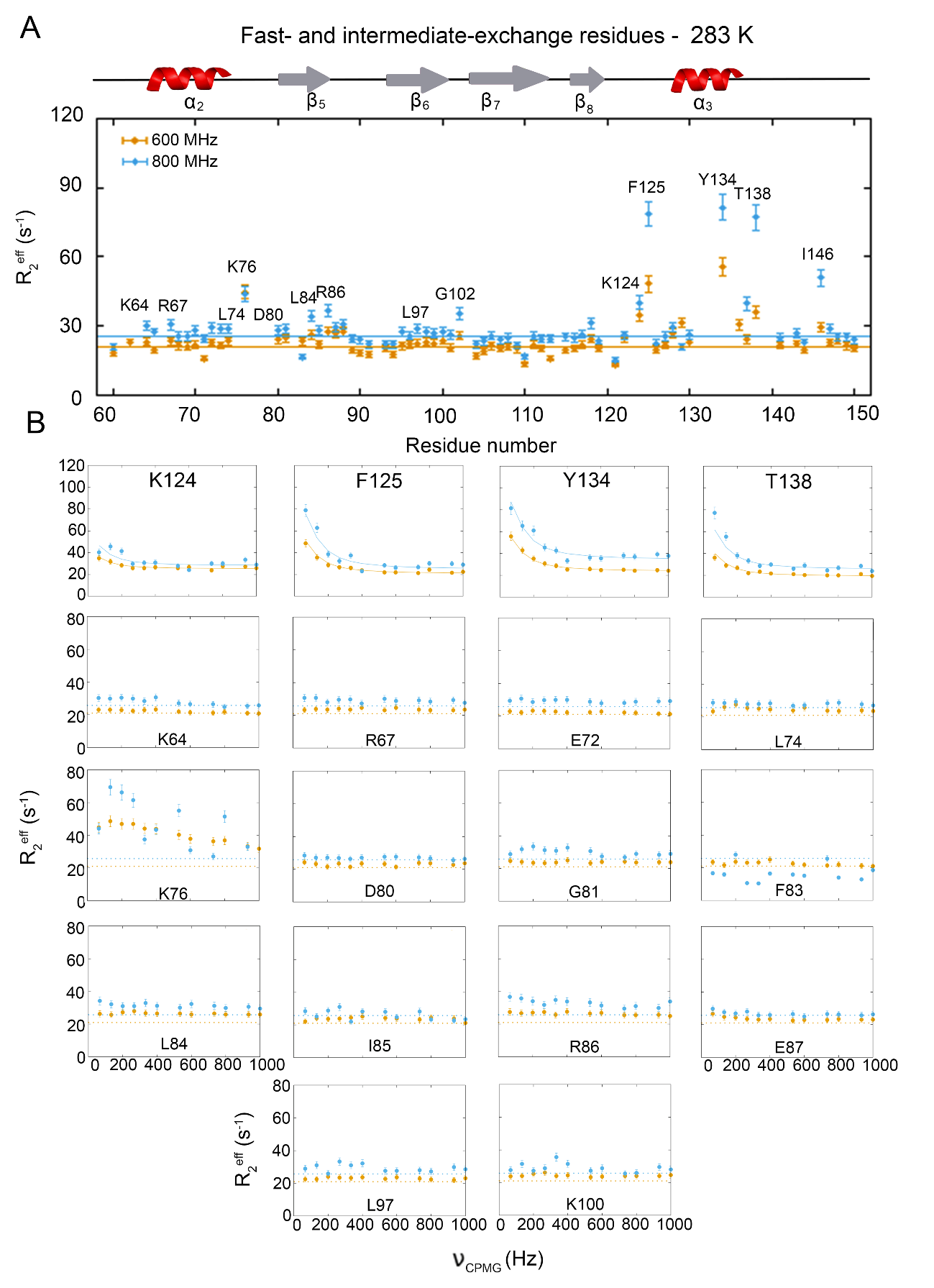


Figure S4: ^15^N CPMG relaxation dispersion profiles (R_2_*^eff^* x ν_CPMG_) at 283 K (10 ºC), pH 7.0. (A) R_2_*^eff^* at 66.7 s^-1^ as a function of the residue number in two fields, 14.09 (600 MHz) and 18.8 T (800 MHz). Different dynamic regions of the protein can be distinguished based on the relaxation: (i) subdomain I: residues in fast conformational exchange (Figure 2A) and (ii) subdomain II: residues in intermediate conformational exchange. The lines show the most likely R_2_*^eff^* without exchange contribution for each dynamic region (R_2_*^eff^*∞). It is used as a reference to determine residues clearly involved in conformational exchange (labelled). The typical ^15^N CPMG-RD profile observed fast exchange regimes is where all the points are above the line, meaning that much larger values of ν_CPMG_ would be necessary to reach R_2_*^eff^*∞. The intermediate exchange regime is characterized by the dispersion curve tending to R_2_*^eff^*∞. The ν_CPMG_ is enough to refocus the conformational exchange and to reach R_2_*^eff^*∞. (B) Selected relaxation dispersion profiles of residues in conformational exchange. K124, F125, Y134, and T138 are in intermediate exchange, and found at subdomain II. We also had a good relaxation dispersion profile for R136 (intermediate exchange) at the 600 MHz. The resonance of R136 at the 800 MHz was not used because it was overlapped with a folded side-chain peak. K64, R67. E72, L74, K76, D80, G81, F83, L84, I85, R86, E87, L97, and K100 are in fast exchange, and found at subdomain II. The orange symbols correspond to the relaxation dispersion obtained at 14.09 T and the blue symbols to the values at 18.8 T.


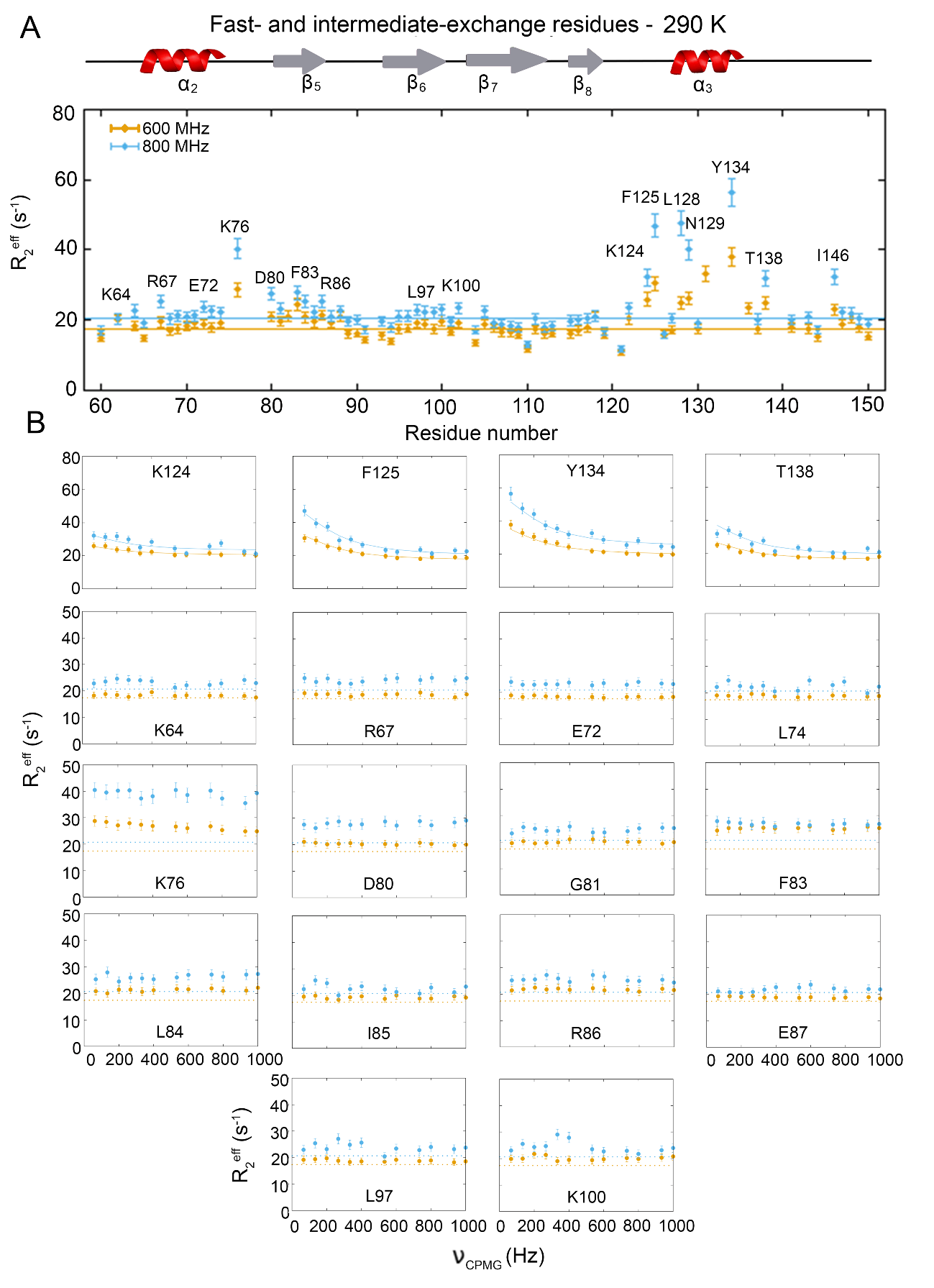


Figure S5: ^15^N CPMG relaxation dispersion profiles (R_2_*^eff^* x ν_CPMG_) at 290 K (17 ºC), pH 7.0. (A) R_2_*^eff^* at 66.7 s^-1^ as a function of the residue number in two fields, 14.09 (600 MHz) and 18.8 T (800 MHz). Different dynamic regions of the protein can be distinguished based on the relaxation: (i) subdomain I: residues in fast conformational exchange (Figure 2A) and (ii) subdomain II: residues in intermediate conformational exchange. The lines show the most likely R_2_*^eff^*  without exchange contribution for each dynamic region (R_2_*^eff^*∞). It is used as a reference to determine residues clearly involved in conformational exchange (labelled). The typical ^15^N CPMG-RD profile observed fast exchange regimes is where all the points are above the line, meaning that much larger values of ν_CPMG_ would be necessary to reach R_2_*^eff^*∞. The intermediate exchange regime is characterized by the dispersion curve tending to R_2_*^eff^*∞. The ν_CPMG_ is enough to refocus the conformational exchange and to reach R_2_*^eff^* ∞. (B) Selected relaxation dispersion profiles of residues in conformational exchange. K124, F125, Y134, and T138 are in intermediate exchange, and found at subdomain II. We also had a good relaxation dispersion profile for R136 (intermediate exchange) at the 600 MHz. The resonance of R136 at the 800 MHz was not used because it was overlapped with a folded side-chain peak. K64, R67. E72, L74, K76, D80, G81, F83, L84, I85, R86, E87, L97, and K100 are in fast exchange, and found at subdomain II. The orange symbols correspond to the relaxation dispersion obtained at 14.09 T (600 MHz) and the blue symbols to the values at 18.8 T (800 MHz).


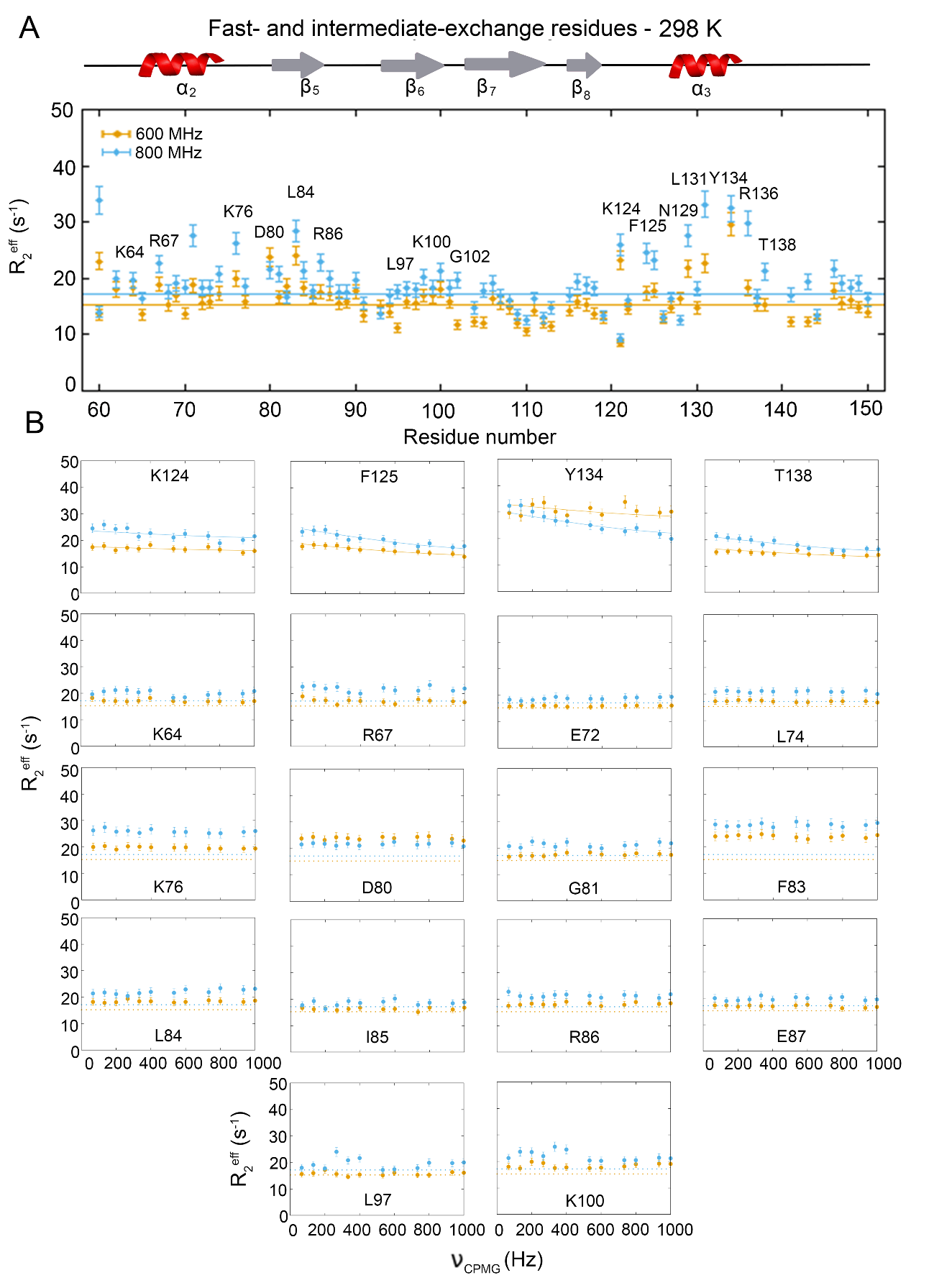


Figure S6: ^15^N CPMG relaxation dispersion profiles (R_2_^eff^ x ν_CPMG_) at 298 K (25 ºC), pH 7.0. (A) R_2_*^eff^* at 66.7 s^-1^ as a function of the residue number in two fields, 14.09 (600 MHz) and 18.8 T (800 MHz). Different dynamic regions of the protein can be distinguished based on the relaxation: (i) subdomain I: residues in fast conformational exchange (Figure 2A) and (ii) subdomain II: residues in intermediate conformational exchange. The lines show the most likely R_2_*^eff^* without exchange contribution for each dynamic region (R_2_*^eff^*∞). It is used as a reference to determine residues clearly involved in conformational exchange (labelled). The typical ^15^N CPMG-RD profile observed fast exchange regimes is where all the points are above the line, meaning that much larger values of ν_CPMG_ would be necessary to reach R_2_*^eff^*∞. The intermediate exchange regime is characterized by the dispersion curve tending to R_2_*^eff^*∞. The ν_CPMG_ is enough to refocus the conformational exchange and to reach R_2_*^eff^*∞. (B) Selected relaxation dispersion profiles of residues in conformational exchange. K124, F125, Y134, and T138 are in intermediate exchange, and found at subdomain II. We also had a good relaxation dispersion profile for R136 (intermediate exchange) at the 600 MHz. The resonance of R136 at the 800 MHz was not used because it was overlapped with a folded side-chain peak. K64, R67. E72, L74, K76, D80, G81, F83, L84, I85, R86, E87, L97, and K100 are in fast exchange, and found at subdomain II. The orange symbols correspond to the relaxation dispersion obtained at 14.09 T (600 MHz) and the blue symbols to the values at 18.8 T (800 MHz).


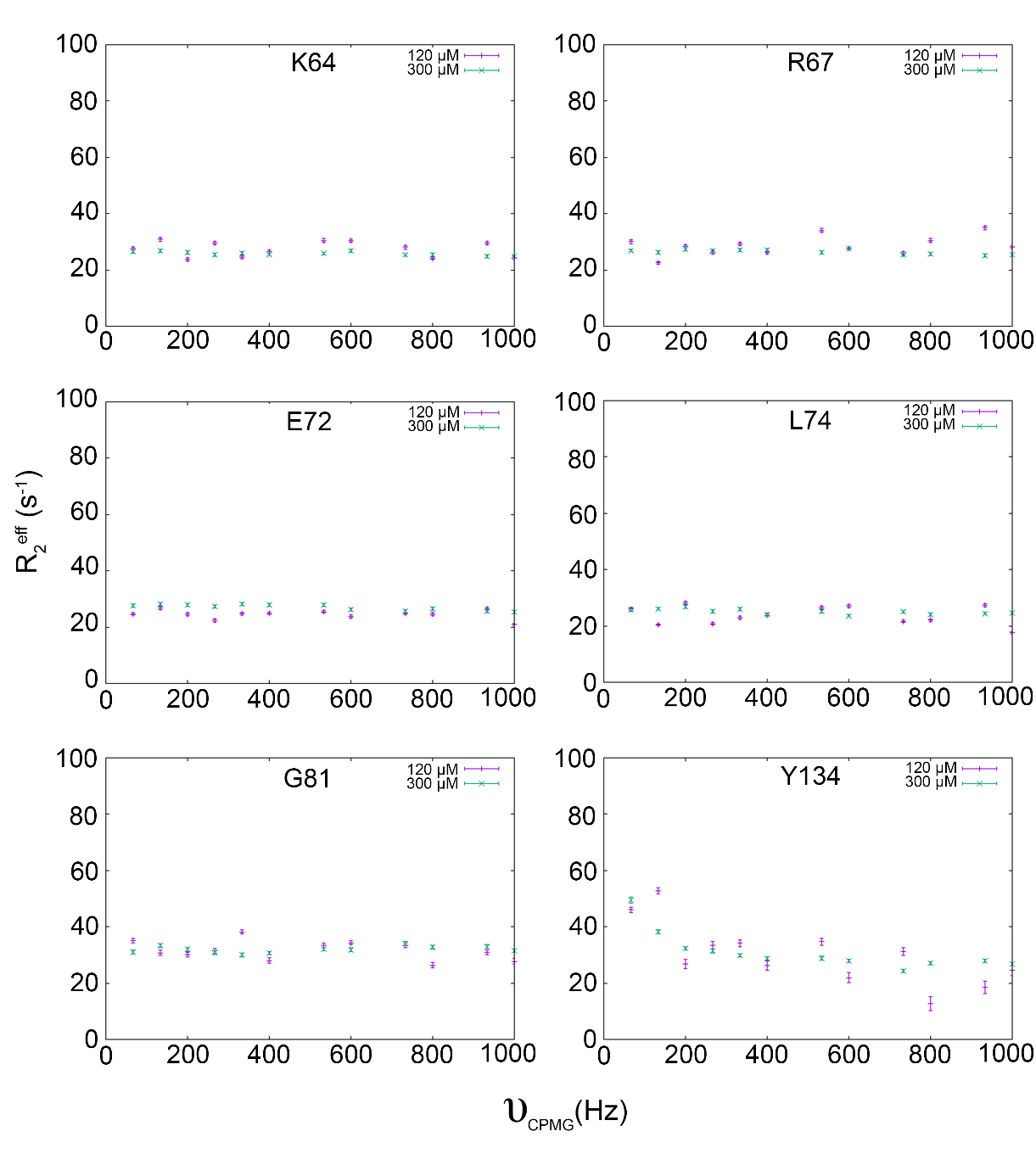


Figure S7: ^15^N CPMG-RD profiles of selected residues (R_2_*^eff^* x ν_CPMG_) at 278 K (5 ºC), pH 7.0 at two concentrations, 120 μM (purple) and 300 μM (green) both acquired at 14.07 T (800 MHz). This is representative of a concentration-independent behavior of ^15^N CPMG-RD. Most of the selected residues are of subdomain I, in fast exchange regime. They have a good signal/noise ratio even at 120 μM. Among the residues of subdomain II, only Y134 yielded a good signal/noise ratio. K124, F125, and T138 have very poor ^15^N CPMG-RD profiles at 120 μM.


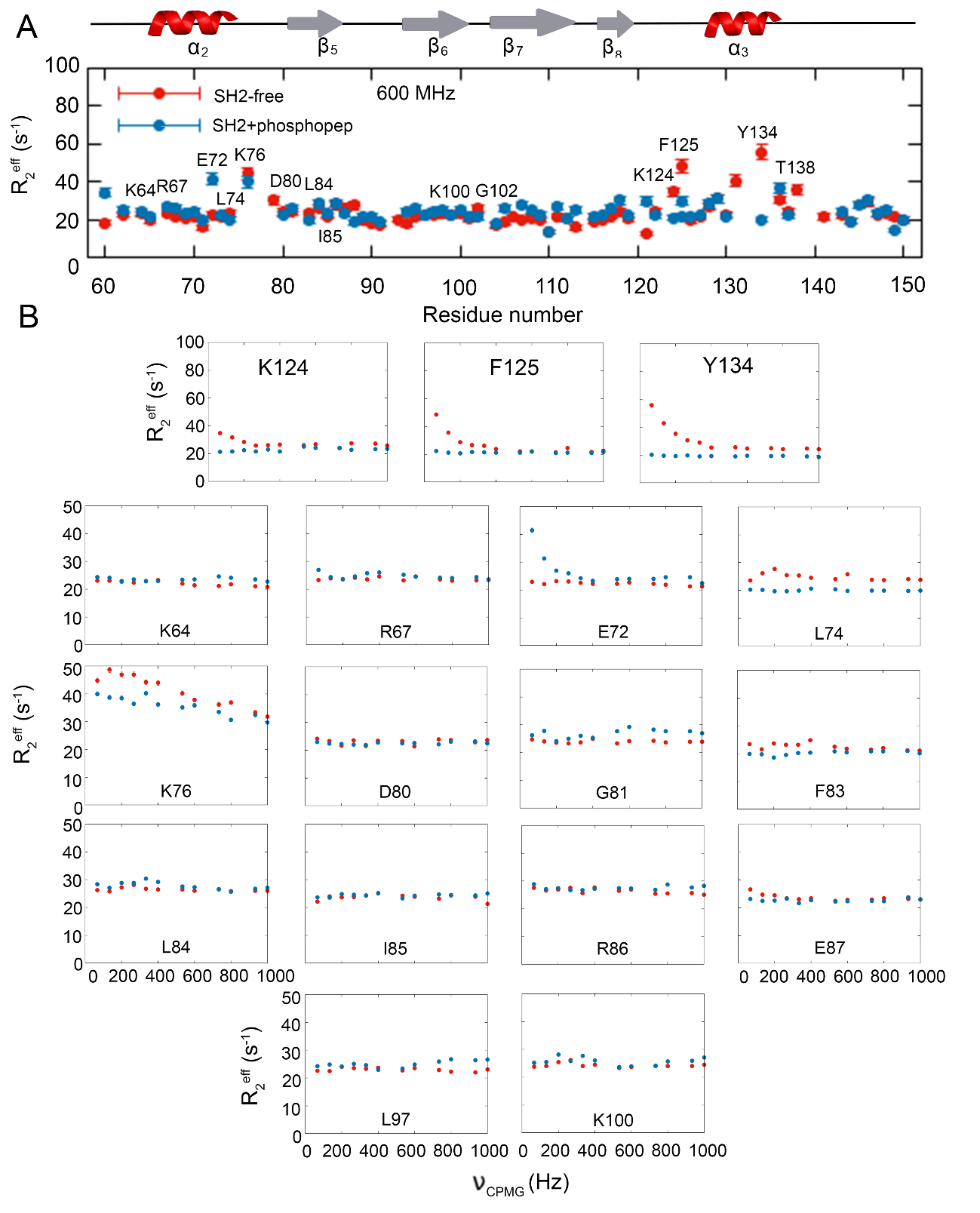


Figure S8: ^15^N CPMG relaxation dispersion profiles (R_2_^eff^ x ν_CPMG_) at 283 K (10 ºC), pH 7.0. (A) R_2_*^eff^* at 66.7 s^-1^ as a function of the residue number at 14.09 T in the presence (blue) and absence (red) of pY-pep. (B) Selected relaxation dispersion profiles of residues in conformational exchange. K124, F125, Y134, and T138 are in intermediate exchange, found at subdomain II. K64, R67. E72, L74, K76, D80, G81, F83, L84, I85, R86, E87, L97, and K100 are in fast exchange, found at subdomain II.


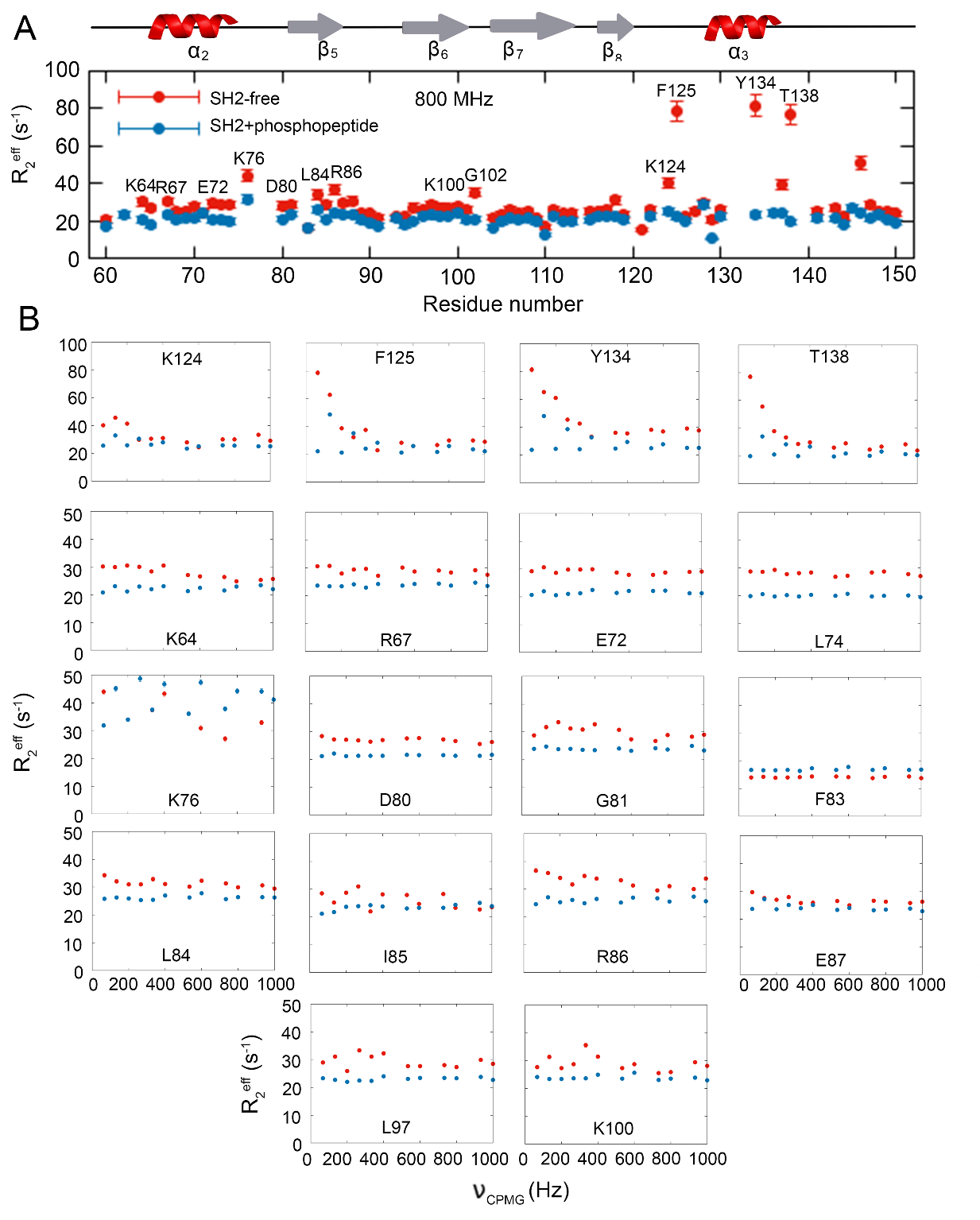


Figure S9: ^15^N CPMG relaxation dispersion profiles (R_2_^eff^ x ν_CPMG_) at 283 K (10 ºC), pH 7.0. (A) R_2_*^eff^* at 66.7 s^-1^ as a function of the residue number at 18.8 T (800 MHz) in the presence (blue) and absence (red) of pY-pep. (B) Selected relaxation dispersion profiles of residues in conformational exchange. K124, F125, Y134, and T138 are in intermediate exchange, found at subdomain II. K64, R67. E72, L74, K76, D80, G81, F83, L84, I85, R86, E87, L97, and K100 are in fast exchange, found at subdomain II.


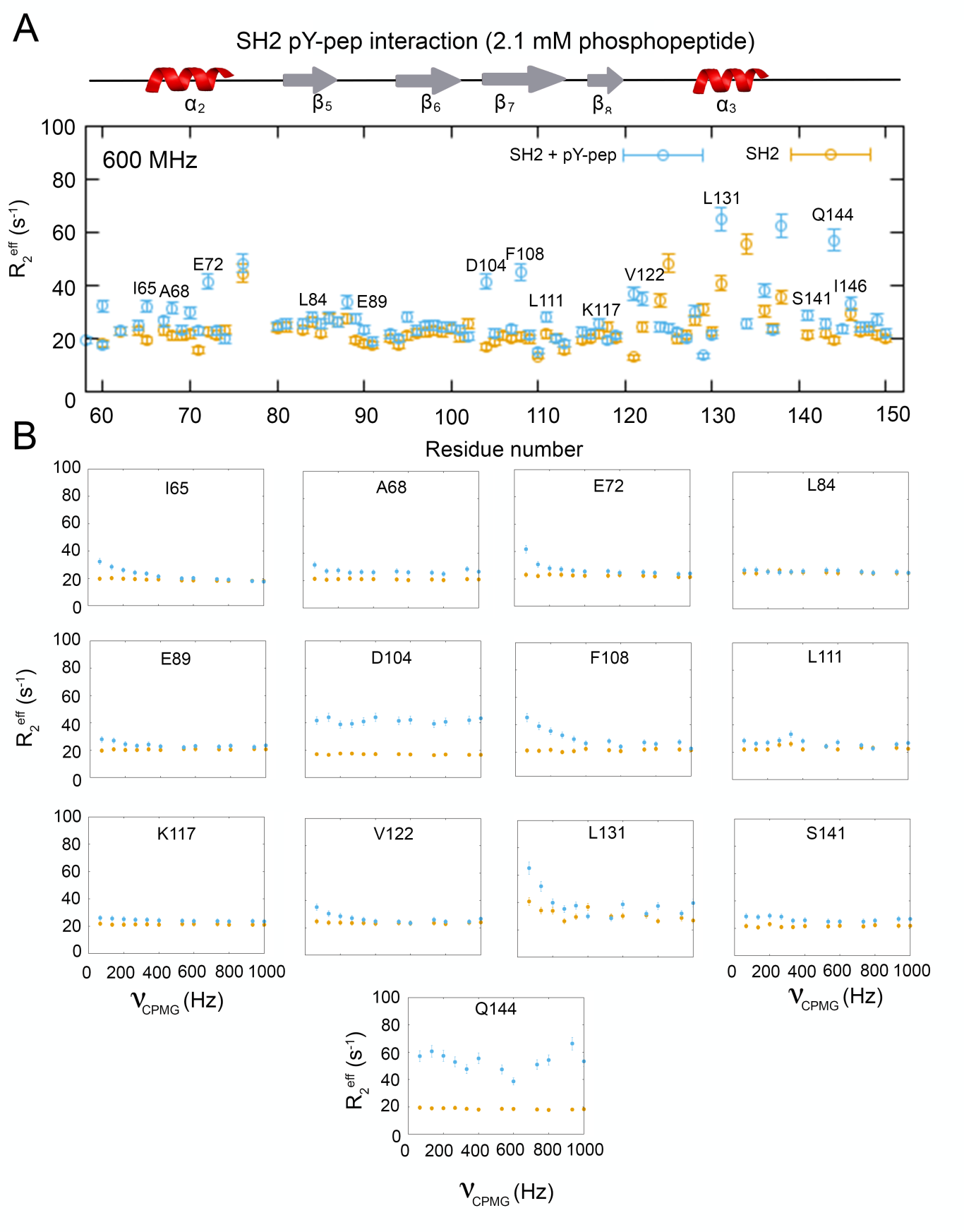


Figure S10: ^15^N CPMG-RD profiles (R_2_^eff^ x ν_CPMG_) at 283 K (10 ºC), pH 7.0, acquired at 14.04 T (600 MHz). (A) R_2_*^eff^* at 66.7 s^-1^ as a function of the residue number in the presence (blue) and absence (orange) of semi-saturated concentration of pY-pep (2.1 mM). (B) Selected relaxation dispersion profiles showing the residues that acquired dispersion in the presence of a semi-saturated concentration of pY-pep. I65, A68, E72, E89, F108, K117, V122, and L131 are in intermediate exchange regime in the presence of pY-pep, while L84, D104, L111, S141, and Q144 are in fast exchange regime.


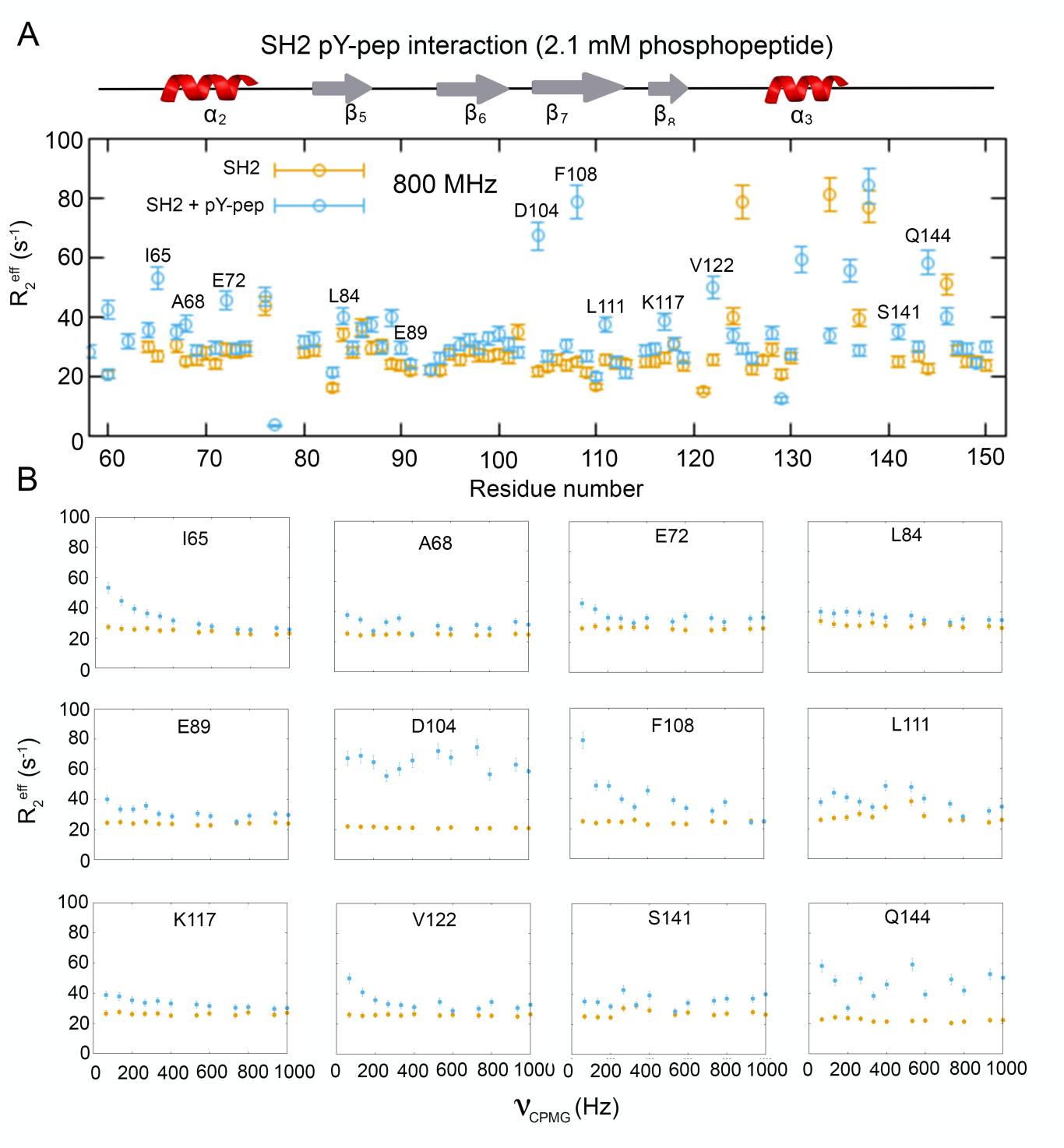


Figure S11: ^15^N CPMG-RD profiles (R_2_^eff^ x ν_CPMG_) at 283 K (10 ºC), pH 7.0 acquired at 18.8 T (800 MHz). (A) R_2_*^eff^* at 66.7 s^-1^ as a function of the residue number. in the presence (blue) and absence (orange) of semi-saturated concentration of pY-pep (2.1 mM). (B) Selected relaxation dispersion profiles showing the residues that acquired dispersion in the presence of a semi-saturated concentration of pY-pep. I65, A68, E72, E89, F108, K117, V122 are in intermediate exchange regime in the presence of pY-pep, while L84, D104, L111, K117, S141, and Q144 are in fast exchange regime.


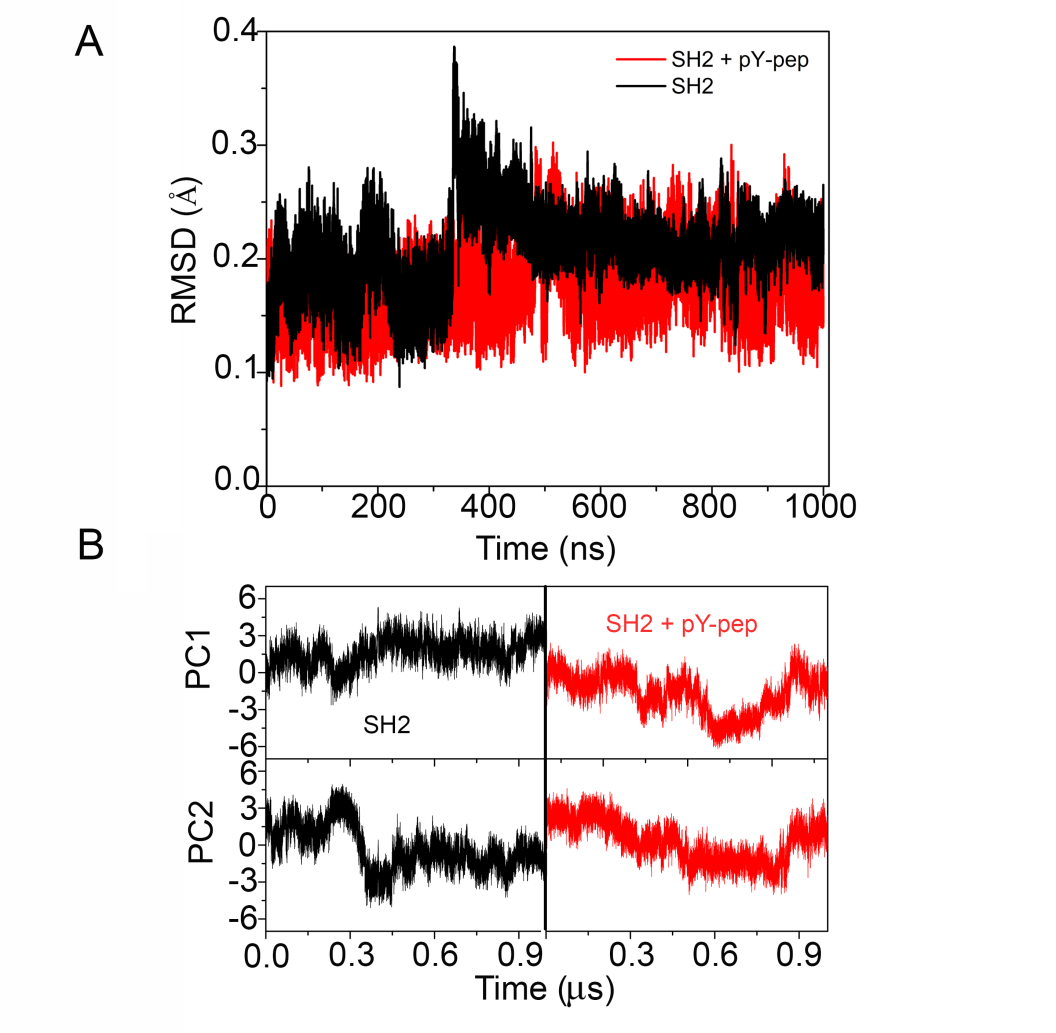


Figure S12: Full-atom molecular dynamic simulation of Grb2-SH2 domain. (A) RMSD of Grb2-SH2 in the presence (red) and absence (black) of pY-pep. (B) Principal eigenvector (PC1) and second principal eigenvector (PC2) for Grb2-SH2 in the presence and absence of pY-pep as a function of the MD simulation time.


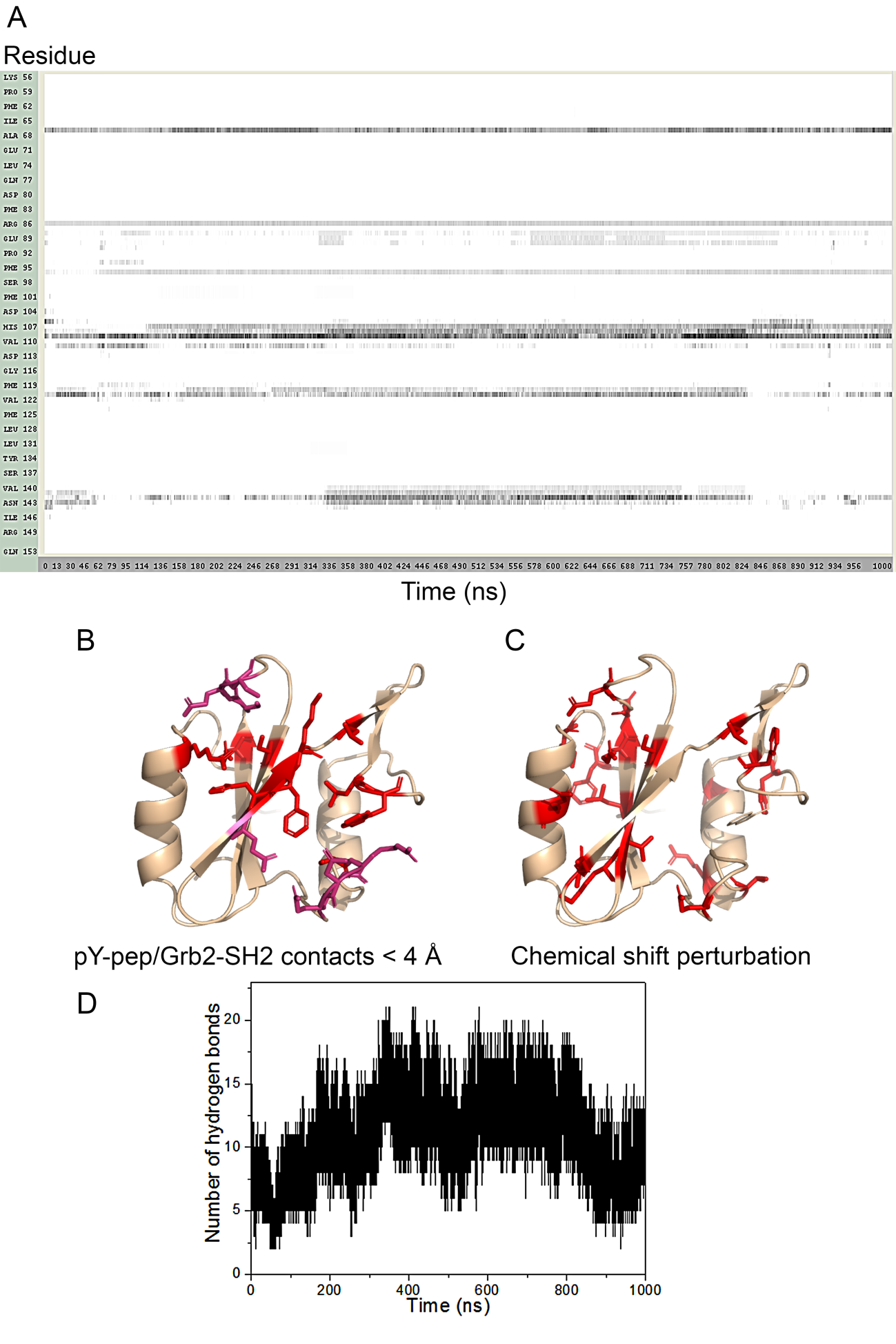


Figure S13. Grb2-SH2 domain/pY-pep contact map. A) Contact of any atom of a given residue of Grb2-SH2 (< 4 Å) with any atom of pY-pep as a function of the simulation time. The color code (from light grey to black) shows the number of contacts of a given residue. B) Ribbon representation of Grb2-SH2 colored according the contacts showed in A. In red are the persistent contacts and in magenta are the intermittent contacts. Note that the persistent contacts are in the center of the recognition face, in accordance with the atomistic density of pY-pep during MD simulation (Figure 6B). C) Ribbon representation of the chemical shift perturbation as showed in Figure 4A. Note that there is a good correspondence between the contacts observed in B and the chemical shift perturbation. D) Number of hydrogen bonds between Grb2-SH2 and pY-pep as a function of the simulation time. The average number of hydrogen bonds is 12.


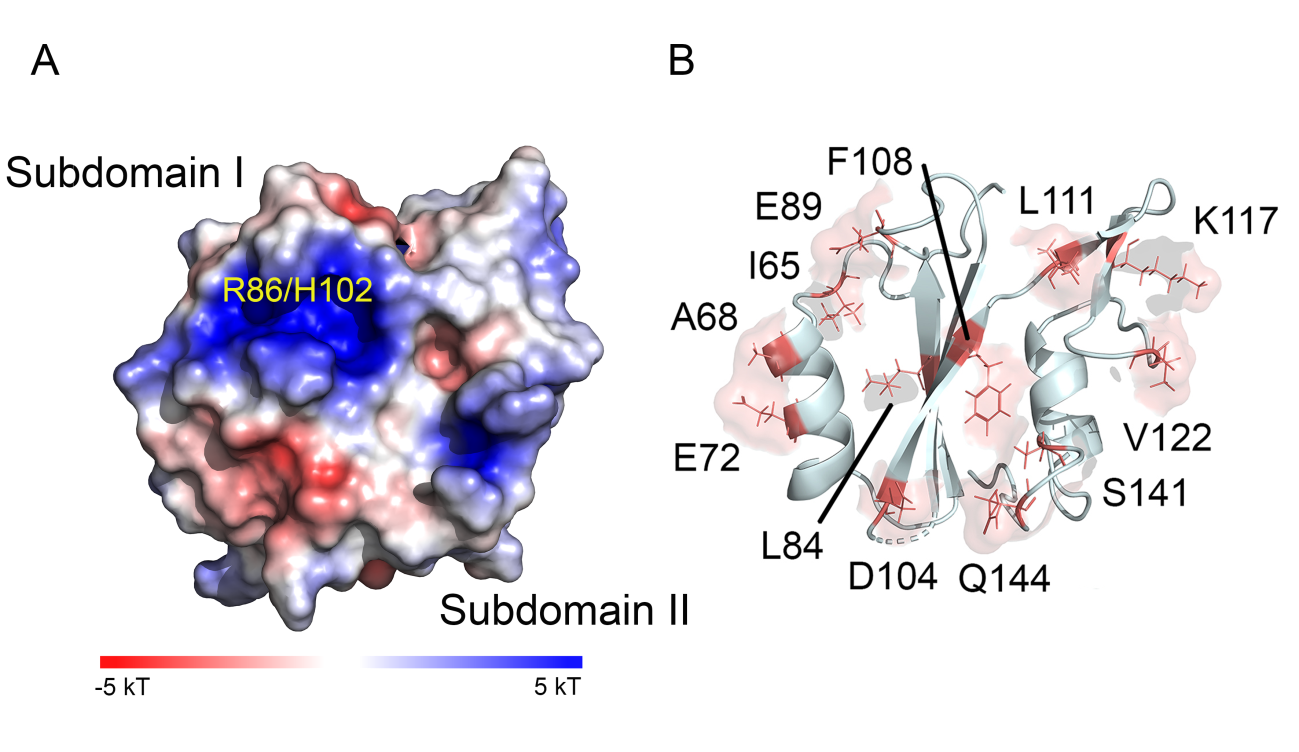


Figure S14: Wide phosphopeptide recognition face of Grb2-SH2. (A) The electrostatic surface and (B) the residues participating in the encounter complex. Note that the recognition surface is much wider than the binding site characterized by the R86/H102 for pY. Electrostatic potential surface of Grb2-SH2 was calculated from APBS software^15^ using charge values and protonation states determined by PDB2PQR server^16^ along with PROPKA program^17^ (pH 7.0, 200 mM NaCl, 25 °C). The bar denotes the electrostatic potential range from –5 (red) to +5 kT (blue). The residues that took part in exchange processes are uniquely at the pY-pep molecular recognition face.

Table S1: Structural statistics for Grb2-SH2 domain

| **Structural statistics for Grb2-SH2** | |
| --- | --- |
| **Number of experimental restraints** |  |
| Total number of calculated structures | 66317 |
| Total NOE distance restrains (ambiguous) | 25 |
| Total NOE distance restrains (unambiguous) | 454 |
| **Ambiguous** | |
| Short range (\|I – j\| = 1) | 7 |
| Medium range (2 ≤ \|I – j\| ≤ 4) | 7 |
| Long range (\|I – j\| > 4) | 11 |
| **Unambiguous** | |
| Short range (\|I – j\| =1) | 159 |
| Medium range (2 ≤ \|I – j\| ≤ 4) | 115 |
| Long range (\|I – j\| > 4) | 180 |
| **RMSD from average structure (Å)** | |
| Backbone (8-96) | 0.502 |
| Backbone, all residues | 0.688 |
| Heavy atoms (8-96) | 0.791 |
| Heavy atoms, all residues | 1.033 |
| **Restraints violations** |  |
| NOE violations (> 0.5 Å) | 7 |
| **Ramachandran plot of ordered residues – Procheck (%)** |  |
| Most favored regions | 86.9 |
| Allowed regions | 13.1 |
| Generously allowed | 0 |
| Disallowed | 0 |
| **Ramachandran plot of ordered residues – Molprobity (%)** |  |
| Most favored regions | 97.9 |
| Allowed regions | 2 |
| Disallowed | 0.1 |
| **Rosetta Energy Score** |  |
| E_Totol_ | -38.4 ± 4.1 |
| E_VdW_ | -422 ± 5.1 |
| E_Elec_ | -142 ± 5.8 |
| Enoe | 217 ± 9.0 |

Table S2: Summary of the individual and global fitting of ^15^N CPMG-RD according Bloch-McConnell equation for subdomain II of Grb2-SH2. We used the following residues: K117,Y118, F119, V122, K124, F125, N126, S127, L128, E130, Y134, R136, and T138. We made the individual fitting for each residue at each temperature and the degrees of freedom (DF) and χ^2^ were computed for each residue. We reported in the table the sum of DF and χ^2^ to compare with the global fittings reported for each temperature and for all temperatures in two conditions. The first was without any boundary and the second imposing linearity of the Arrhenius equation. Note that, because of the low number of residues in intermediate conformational exchange at subdomain two (Figures S2, S3, S4, and S5), there was a poor convergence of the population of the minor state (p_B_). The global fitting at all temperatures and especially the imposition of linearity of Arrhenius equation enabled a fairly good convergence of p_B_. We obtained a good convergence for k_ex_ in all situations. These data enabled the computation of the thermodynamic parameters (∆G, ∆H, and ∆S) for the equilibrium and transition state (Figure 3).

| **Individual fits** | | | | | | |
| --- | --- | --- | --- | --- | --- | --- |
| T, ^o^C | | ΣDF | | Σχ^2^ | p_B_, % | k_ex_, s^-1^ |
| 5 | |  | |  |  | 515±237 |
| 10 | |  | |  | * | 557±325 |
| 17 | | 988 | | 662.7 |  | 1374±256 |
| 25 | |  | |  |  | 4006±1236 |
| **Global fit each temperature** | | | | | | |
| T, ^o^C | | DF | | χ^2^ | p_B_, % | k_ex_, s^-1^ |
| 5 | | 239 | | 123 | * | 591±361 |
| 10 | | 260 | | 195 | * | 693±95 |
| 17 | | 260 | | 103 | * | 1486±140 |
| 25 | | 281 | | 138 | * | 4526±1922 |
| **Global fit all temperatures** | | | | | | |
| T, ^o^C | | | DF | χ^2^ | p_B_, % | k_ex_, s^-1^ |
| 5 | | |  |  | 0.17±0.04 | 532±74 |
| 10 | | |  |  | 0.23±0.06 | 649±62 |
| 17 | | | 1057 | 719 | 0.17±0.05 | 1532±131 |
| 25 | | |  |  | 0.16±0.10 | 4407±1307 |
| **Global fit all temperatures imposing Arrhenius linearity** | | | | | | |
| T, ^o^C | DF | | | χ^2^ | p_B_, % | k_ex_, s^-1^ |
| 5 |  | | |  | 0.14±0.03 | 390±54 |
| 10 |  | | |  | 0.13±0.03 | 704±67 |
| 17 | 1061 | | | 746 | 0.11±0.03 | 1559±133 |
| 25 |  | | |  | 0.096±0.06 | 3708±1100 |
| *p_B_ did not converge. | | | | | | |

Table S3: Description of the NMR experiments used for the structural restraints and relaxation dispersion of Grb2-SH2.

| **Experiment** | **Acquisition (number of points in the time domain, acquisition time)** | | **Experimental details** |
| --- | --- | --- | --- |
| **14.09 T, 600 MHz** | | | |
| 15N-CPMG-RD  3 temperatures: 283, 290, 298 K  (300 μM Grb2-SH2) | ^1^H (1024, 53.2480ms), ^15^N (100, 27.4042ms)  pseudo3d: 13 points | | DQD, Echo-AntiEcho |
| 15N-CPMG-RD  278 K  two samples (120 and 300 μM Grb2-SH2) | ^1^H (1024, 53.2480ms), ^15^N (100, 28.3539ms)  pseudo3d: 13 points | | DQD, Echo-AntiEcho |
| 15N-CPMG-RD  283 K  (300 μM Grb2-SH2 with 6.2 mM pY-pep) | ^1^H (1024, 53.2480ms), ^15^N (140, 39.6955ms)  pseudo3d: 13 points | | DQD, Echo-AntiEcho |
| 15N-CPMG-RD  283 K  (300 μM Grb2-SH2 with 2.1 mM pY-pep) | ^1^H (1024, 53.2480ms), ^15^N (140, 39.6955ms)  pseudo3d: 13 points | | DQD, Echo-AntiEcho |
| **18.8 T, 800MHz** | | | |
| 15N-CPMG-RD  3 temperatures: 283, 290, 298 K  (300 μM Grb2-SH2) | | ^1^H (1024, 35.4987ms), ^15^N (100, 19.8845ms)  pseudo3d: 13 points | DQD, Echo-AntiEcho |
| 15N-CPMG-RD  278 K  two samples (120 and 300 μM Grb2-SH2) | | ^1^H (1024, 35.4987ms), ^15^N (100, 19.8845ms)  pseudo3d: 13 points | DQD, Echo-AntiEcho |
| 15N-CPMG-RD  283 K  (300 μM Grb2-SH2 with 6.2 mM pY-pep) | | ^1^H (1024, 35.4987ms), ^15^N (110, 21.8730ms)  pseudo3d: 13 points | DQD, Echo-AntiEcho |
| 15N-CPMG-RD  283 K  (300 μM Grb2-SH2 with 2.1 mM pY-pep) | | ^1^H (1024, 35.4987ms), ^15^N (110, 21.8730ms)  pseudo3d: 13 points | DQD, Echo-AntiEcho |
| 15N-R2 (CPMH)  298 K (300 μM Grb2-SH2) | | ^1^H (1024, 42.5984ms), ^15^N (90, 19.1303ms)  pseudo3d: 6 points | DQD, Echo-AntiEcho |
| 15N-R2 (CPMH) 298 K (300 μM Grb2-SH2) | | ^1^H (1024, 42.5984ms), ^15^N (90, 19.1303ms)  pseudo3d: 6 points | DQD, Echo-AntiEcho |
| Heteronuclear-NOE  (300 μM Grb2-SH2) | | ^1^H (1024, 42.5984ms), ^15^N (90, 19.1303ms)  saturation time = 6 s | DQD, Echo-AntiEcho |
| **21.1 T, 900MHz** | | | |
| ^13^C NOESY-HSQC | ^1^H (1024, 40.5504 ms), ^13^C (80, 4.4176 ms), ^1^H (184, 7.2864 ms)  τm = 100 ms | | DQD, Echo-AntiEcho, State-TPPI 40% NUS point (aliphatic NOESY) |
| ^13^C NOESY-HSQC | ^1^H (1024, 40.5504 ms), ^13^C (64, 4.7121 ms), ^1^H (180, 7.1280 ms) τm = 100 ms | | DQD, Echo-AntiEcho, State-TPPI 40% NUS point (aromatic NOESY) |
| ^15^N NOESY-HSQC | ^1^H (1024, 40.5504 ms), ^15^N (70, 12.7884 ms), ^1^H (180, 7.1280 ms)  τm = 100 ms | | DQD, Echo-AntiEcho, State-TPPI 50% NUS point |

**References**

1. Sanches, K., Caruso, Í. P., Almeida, F. C. L. & Melo, F. A. NMR assignment of free 1H, 15N and 13C-Grb2-SH2 domain. *Biomol. NMR Assign.* (2019). doi:10.1007/s12104-019-09894-x

2. Mao, B., Tejero, R., Baker, D. & Montelione, G. T. Protein NMR structures refined with rosetta have higher accuracy relative to corresponding x-ray crystal structures. *J. Am. Chem. Soc.* **136**, 1893–1906 (2014).

3. Shen, Y. *et al.* Consistent blind protein structure generation from NMR chemical shift data. *Proc. Natl. Acad. Sci. U. S. A.* **105**, 4685–90 (2008).

4. Senior, M. M. *et al.* The Three-Dimensional Solution Structure of the Src Homology Domain-2 of the Growth Factor Receptor-Bound Protein-2. *J. Biomol. NMR* **11**, 153–164 (1998).

5. Thornton, K. H. *et al.* Nuclear Magnetic Resonance Solution Structure of the Growth Factor Receptor-Bound Protein 2 Src Homology 2 Domain. *Biochemistry* **35**, 11852–11864 (1996).

6. Morando, M. A., Barbosa, G. M., Cruz-Oliveira, C., Da Poian, A. T. & Almeida, F. C. L. Dynamics of Zika Virus Capsid Protein in Solution: The Properties and Exposure of the Hydrophobic Cleft Are Controlled by the α-Helix 1 Sequence. *Biochemistry* **58**, 2488–2498 (2019).

7. Cierpicki, T., Zhukov, I., Byrd, R. A. & Otlewski, J. Hydrogen Bonds in Human Ubiquitin Reflected in Temperature Coefficients of Amide Protons. *J. Magn. Reson.* **157**, 178–180 (2002).

8. Hong, J., Jing, Q. & Yao, L. The protein amide ^1^H(N) chemical shift temperature coefficient reflects thermal expansion of the N-H···O=C hydrogen bond. *J. Biomol. NMR* **55**, 71–8 (2013).

9. McConnell, H. M. Reaction Rates by Nuclear Magnetic Resonance. *J. Chem. Phys.* **28**, 430–1 (1958).

10. Korzhnev, D. M. *et al.* Low-populated folding intermediates of Fyn SH3 characterized by relaxation dispersion NMR. *Nature* **430**, 586–90 (2004).

11. Korzhnev, D. M., Religa, T. L., Lundström, P., Fersht, A. R. & Kay, L. E. The folding pathway of an FF domain: characterization of an on-pathway intermediate state under folding conditions by (15)N, (13)C(alpha) and (13)C-methyl relaxation dispersion and (1)H/(2)H-exchange NMR spectroscopy. *J. Mol. Biol.* **372**, 497–512 (2007).

12. Korzhnev, D. M. *et al.* Probing the Transition State Ensemble of a Protein Folding Reaction by Pressure-Dependent NMR Relaxation Dispersion. *J. Am. Chem. Soc.* **128**, 5262–5269 (2006).

13. Choy, W.-Y., Zhou, Z., Bai, Y. & Kay, L. E. An 15N NMR spin relaxation dispersion study of the folding of a pair of engineered mutants of apocytochrome b562. *J. Am. Chem. Soc.* **127**, 5066–72 (2005).

14. Hagen, S. J., Hofrichter, J., Szabo, A. & Eaton, W. A. Diffusion-limited contact formation in unfolded cytochrome c: estimating the maximum rate of protein folding. *Proc. Natl. Acad. Sci.* **93**, 11615–11617 (1996).

15. Baker, N. A., Sept, D., Joseph, S., Holst, M. J. & McCammon, J. A. Electrostatics of nanosystems: Application to microtubules and the ribosome. *Proc. Natl. Acad. Sci. U. S. A.* **98**, 10037–10041 (2001).

16. Dolinsky, T. J., Nielsen, J. E., McCammon, J. A. & Baker, N. A. PDB2PQR: An automated pipeline for the setup of Poisson-Boltzmann electrostatics calculations. *Nucleic Acids Res.* **32**, (2004).

17. Biswas-Fiss, E. E., Kukiratirat, J. & Biswas, S. B. Thermodynamic analysis of DNA binding by a Bacillus single stranded DNA binding protein. *BMC Biochem.* **13**, (2012).
